# Does government-assisted relocation help households

**DOI:** 10.1126/sciadv.aef4602

**Published:** 2026-11-11

**Authors:** Rachel Young

**Affiliations:** Humphrey School of Public Affairs, University of Minnesota Twin Cities, 301 S 19th Ave, Minneapolis, MN 55455, USA.

## Abstract

Climate change is intensifying floods and storms, leading governments to consider relocating households away from high-risk areas. Yet, there is little evidence on whether such programs reduce risk or improve household well-being. I evaluate the US Federal Emergency Management Agency’s property acquisition (“buyout”) program using nationwide longitudinal records that track households before and after approval. By comparing households facing similar mapped flood risks, I estimate the program’s effects on relocation, residence in high-risk flood areas, and financial outcomes. Households that participate are more likely to relocate outside Federal Emergency Management Agency–designated flood hazard areas and experience short-term reductions in debt and improvements in neighborhood socioeconomic conditions. However, these benefits are concentrated among participants from wealthier and predominantly white neighborhoods, who relocate more quickly and achieve larger reductions in risk. A benefit-cost analysis shows that the program value depends on implementation costs and realized reductions in flood-risk exposure, with benefit-cost ratios ranging from 0.84 to 1.28 across three scenarios. These results show that government-assisted relocation can be an effective adaptation tool, but its social and economic benefits remain unevenly distributed.

## INTRODUCTION

Climate change is forcing societies to confront how and where people can safely live. One emerging approach is not to defend every place but to help people and assets move away from areas of growing environmental risk. This strategy is known as managed retreat or government-supported relocation (*1*). Around the world, governments are expanding such programs, for example, coastal resettlement in the Philippines (*2*) and Indonesia (*3*) and floodplain resettlement schemes in Vietnam (*4*). As climate change intensifies floods, fires, and storms, these programs are becoming an increasingly prominent yet controversial adaptation tool. A growing body of research has described elements of managed retreat in the US (*1*, *5*–*7*). Yet, little evidence has been presented to answer the basic question: Can we help people move out of harm’s way without making them worse off?

In the US, the Federal Emergency Management Agency (FEMA) has operated a property buyout program for more than three decades, helping households in flood-prone areas permanently relocate. Because few countries operate retreat programs with comparable scale, duration, or administrative structure, FEMA’s experience provides a rare opportunity to evaluate how assisted relocation affects household outcomes and to inform global adaptation policy. However, two challenges have long hindered such evaluations. First, people relocate for many reasons, making it difficult to determine how much relocation and flood-risk reduction can be attributed to government assistance rather than mobility that would have occurred anyway. Second, longitudinal data rarely track household residential locations over long periods, limiting the ability to evaluate how relocation patterns and exposure to flood-prone areas evolve following program participation.

Here, I overcome both challenges by combining administrative FEMA records with commercially sourced individual-level data, tracking households before and after buyout approval and comparing participants with otherwise similar nonparticipants living in the same flood-exposed areas (Fig. 1A). This linked dataset enables a national evaluation of the long-run consequences of government-supported relocation.

**Fig. 1.**
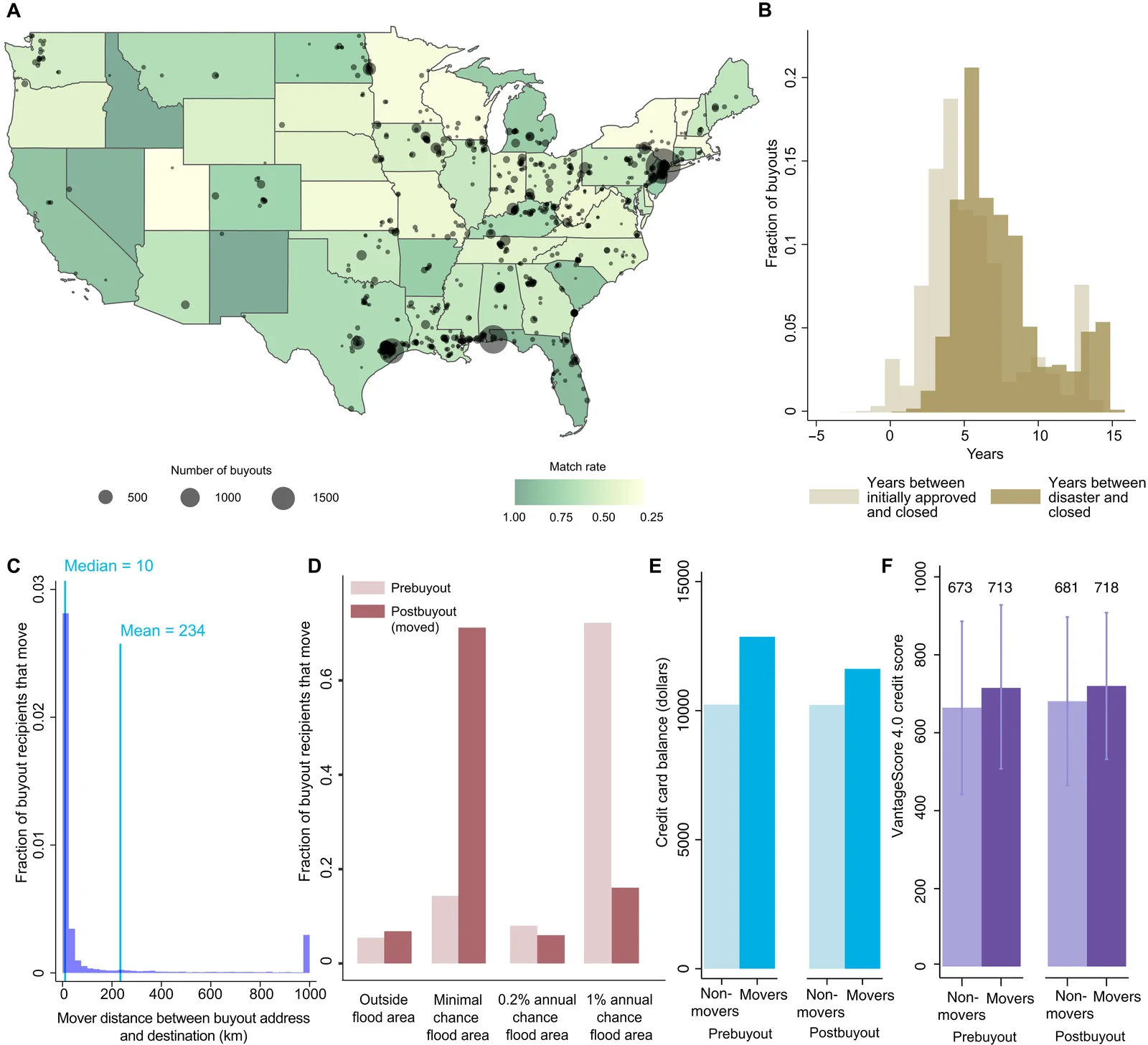
Descriptive information on the buyout participants (treated sample). (**A**) Locations and counts of buyout recipients between 2000 and 2017 in the matched sample (bubble size corresponds to the number of matches in a ZIP code). State-level match rates between Infutor and buyout addresses are shown (dark green = 100% match rate; light green = 25% match rate). Base map: US Census Bureau TIGER/Line Shapefiles (*43*). (**B**) Distribution of the number of years between the start of the buyout process and buyout closure. Dark brown bars show the difference between the disaster year and the closure year; light brown bars show the difference between the approval year and the closure year. (**C**) Distance (km) between the buyout address and the new addresses for buyout participants who move. (**D**) Fraction of matched buyout participants living in the 1% annual-chance flood hazard area (100-year floodplain), the 0.2% annual-chance flood hazard area (500-year floodplain), the minimal flood hazard area, and outside the mapped flood hazard area, both before the buyout (light maroon) and 5 years after the buyout, conditional on moving (dark maroon). (**E**) Average credit card balances (dollars) for buyout participants 1 year before the buyout (“Prebuyout”) and 5 years after (“Postbuyout”), shown separately for movers and nonmovers. Differences are not statistically significant. (**F**) Average VantageScore 4.0 credit scores for buyout participants 1 year before (“Prebuyout”) and 5 years after (“Postbuyout”), shown separately for movers and nonmovers. Capped bars indicate 95% confidence intervals; mean values are printed above bars.

Ideally, we would observe two identical households—one receiving a government-funded buyout and one not—and follow their outcomes over time. Such an experiment is impossible, but the assembled dataset allows for comparisons between buyout recipients and households with comparable mapped flood-risk exposure who did not participate in the program. Using this design, I estimate the effects of program participation on residential mobility, map flood exposure and financial well-being, and combine these estimates with federal spending data to evaluate the program’s fiscal cost-effectiveness.

The analysis reveals three main findings. Buyout participation substantially increases the likelihood of residing outside FEMA-designated high-risk flood areas, increases relocation to higher-income neighborhoods, and is not associated with persistent deterioration in observed financial outcomes. These findings help explain the benefit-cost analysis results. Because the program increases the likelihood of residing outside of the FEMA-mapped and designated “Special Flood Hazard Area” (SFHA) by less than would be implied by assuming that all observed moves are program induced, the estimated benefits are substantially reduced once implementation delays and incomplete additionality are taken into account. As a result, fiscal performance depends critically on both program administration and the extent to which relocation outcomes exceed those that would have occurred absent participation.

### How the FEMA buyout program works

The FEMA property acquisition (“buyout”) program aims to reduce future flood losses by purchasing damaged homes at predisaster market value and converting the land to a permanent open space (fig. S1) (*8*, *9*). Since its inception in 1989, FEMA has funded more than 53,000 buyouts across all US states (Fig. 1A and fig. S2), making it the nation’s largest managed-retreat initiative (*10*). The program is designed primarily to reduce property risk and federal disaster spending, but its effects on participating households remain largely unknown because FEMA does not track households after they sell their homes.

Participation in the program is decentralized and shaped by multiple stages of selection. Following a federally declared disaster, local governments propose buyout projects to state agencies and FEMA, which evaluate applications using criteria including flood risk, expected future damages, cost-effectiveness, and prior disaster impacts (*8*). As a result, households receiving buyout approvals are likely to differ systematically from other households in flood-prone areas, particularly in recent flood exposure and damage severity. At the same time, the buyout process is also complex and slow, averaging more than 6 years from disaster declaration to project closure (Fig. 1B). These implementation realities suggest that real-world performance may differ substantially from theoretical expectations or idealized planning timelines and underscore the importance of evaluating how households actually relocate and fare following program approval.

### Identification and interpretation

Individuals relocate for many reasons (*11*–*14*), and residents of buyout areas differ systematically from the general population (fig. S3A) (*1*, *5*). To estimate the program’s effects while improving comparability between treated and untreated households, I implement a difference-in-differences design comparing buyout recipients with nonparticipants who lived in the same ZIP code and within the SFHAs (see Materials and Methods). While the SFHA is an imperfect physical measure of risk, it is a clear regulatory indicator for property owners living in high-risk floodplains. Restricting comparisons to neighboring households within SFHAs improves pretreatment balance across a broad set of observable household characteristics, including financial measures and mobility indicators, as well as neighborhood demographics (tables S2 and S3). Additional analyses within finer flood-risk categories yield qualitatively similar results and improved balance across individual indicators (fig. S4 and table S3). In addition, treated and untreated households exhibit highly similar pretreatment trends across the main outcomes (Fig. 2 and figs. S3 to S6), supporting the use of these comparison groups for evaluating program impacts while recognizing that selection on unobservable characteristics and nonadditionality remain possible. The design cannot rule out selection on the basis of contemporaneous flood damage, which may independently influence both relocation and subsequent flood-risk exposure (see Materials and Methods).

**Fig. 2.**
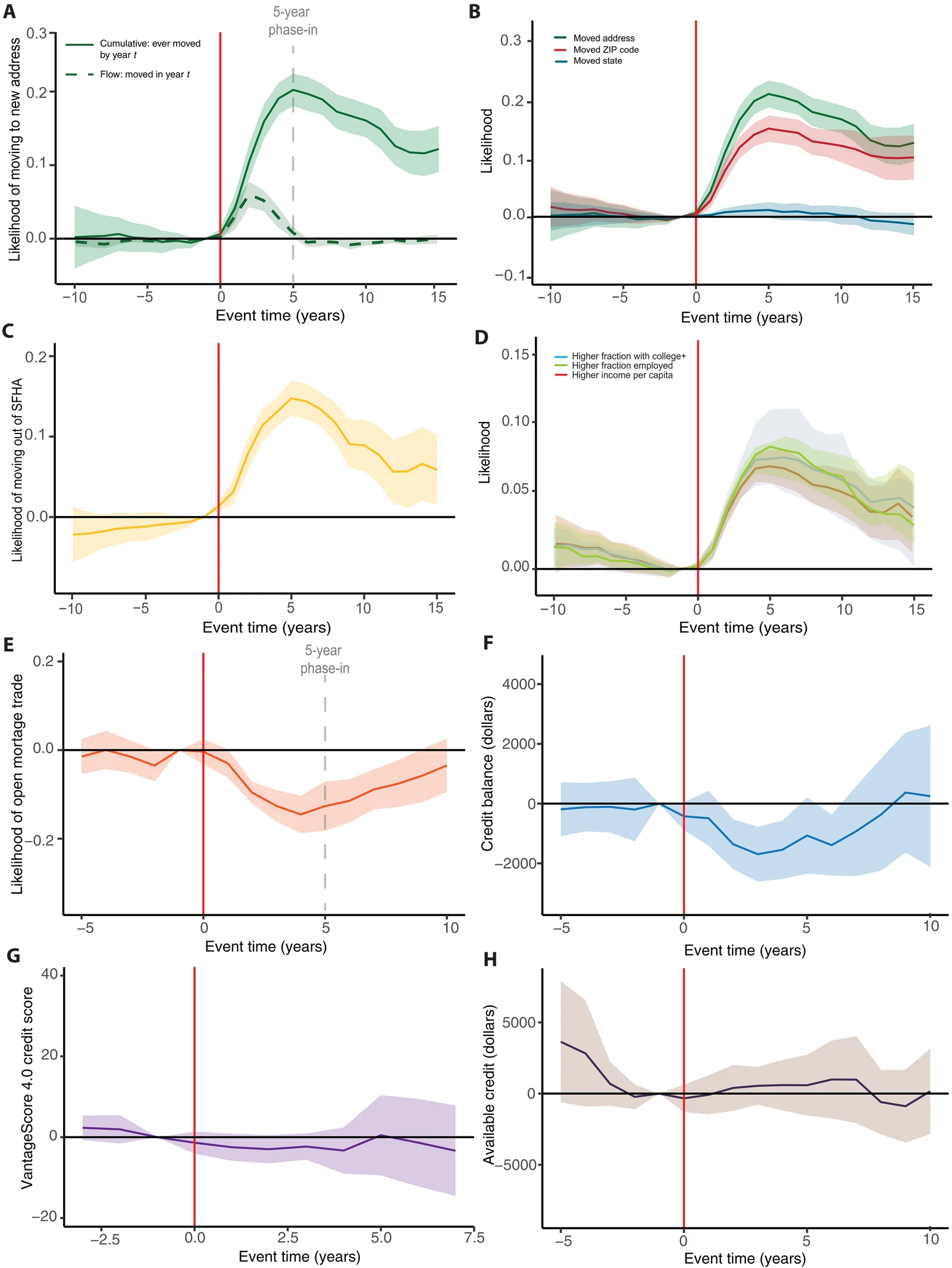
Effect of the buyout on individuals’ locations and financial outcomes. (**A**) Estimated cumulative effect, with 95% confidence intervals, of participation in the buyout program (2000 to 2017) on relocation to a new address. The figure reports cumulative sums of the event-time coefficients estimated in Eq. 2, plotted from 10 years before to 15 years after buyout approval, and corresponding period-specific (flow) effects on annual relocation estimated in Eq. 1. (**B** to **D**) Same as (A) but for (B) moving to a new ZIP code (red) or a new state (blue), (C) moving out of an SFHA, and (D) moving to census tracts with higher educational attainment (blue), higher employment (green), or higher income (red). (**E** to **H**) Effects of participating in the buyout program (2008 to 2017) on credit report outcomes: (E) number of mortgages, (F) total credit balance, (G) VantageScore 4.0 credit score (estimated for 2010 to 2017), and (H) available credit.

I interpret the main estimates as reflecting the program’s real-world performance, including long processing times, incomplete participation, and households that ultimately opt out (Materials and Methods, Fig. 1B, and figs. S3 and S5). Because not all approved households relocate, and those who do often move years after approval (*15*), these estimates capture the program’s effects under observed implementation conditions. Moreover, because untreated households facing similar flood-risk exposure also relocate over time, the estimated treatment effects capture the incremental relocation and flood-risk reduction associated with buyout participation beyond mobility that would have occurred in the absence of the program.

To assess broader financial implications, I examine four indicators commonly used in studies of economic mobility and credit access—mortgage holding, credit-card debt, available credit, and overall credit score (Figs. 1, E and F, and 2, E to H, and fig. S6) (*16*, *17*). These outcomes provide complementary evidence on housing stability, borrowing behavior, and financial resilience.

Last, because equitable adaptation is a central policy concern, I analyze heterogeneity in program impacts across communities. Past research suggests that lower-income and racially diverse neighborhoods face higher administrative burdens and lower participation in hazard-mitigation programs (*5*–*7*, *18*, *19*). I test whether participants from lower-income or majority-Black census tracts experience similar reductions in flood exposure and similar financial outcomes as those from more advantaged areas (Fig. 3 and fig. S7).

**Fig. 3.**
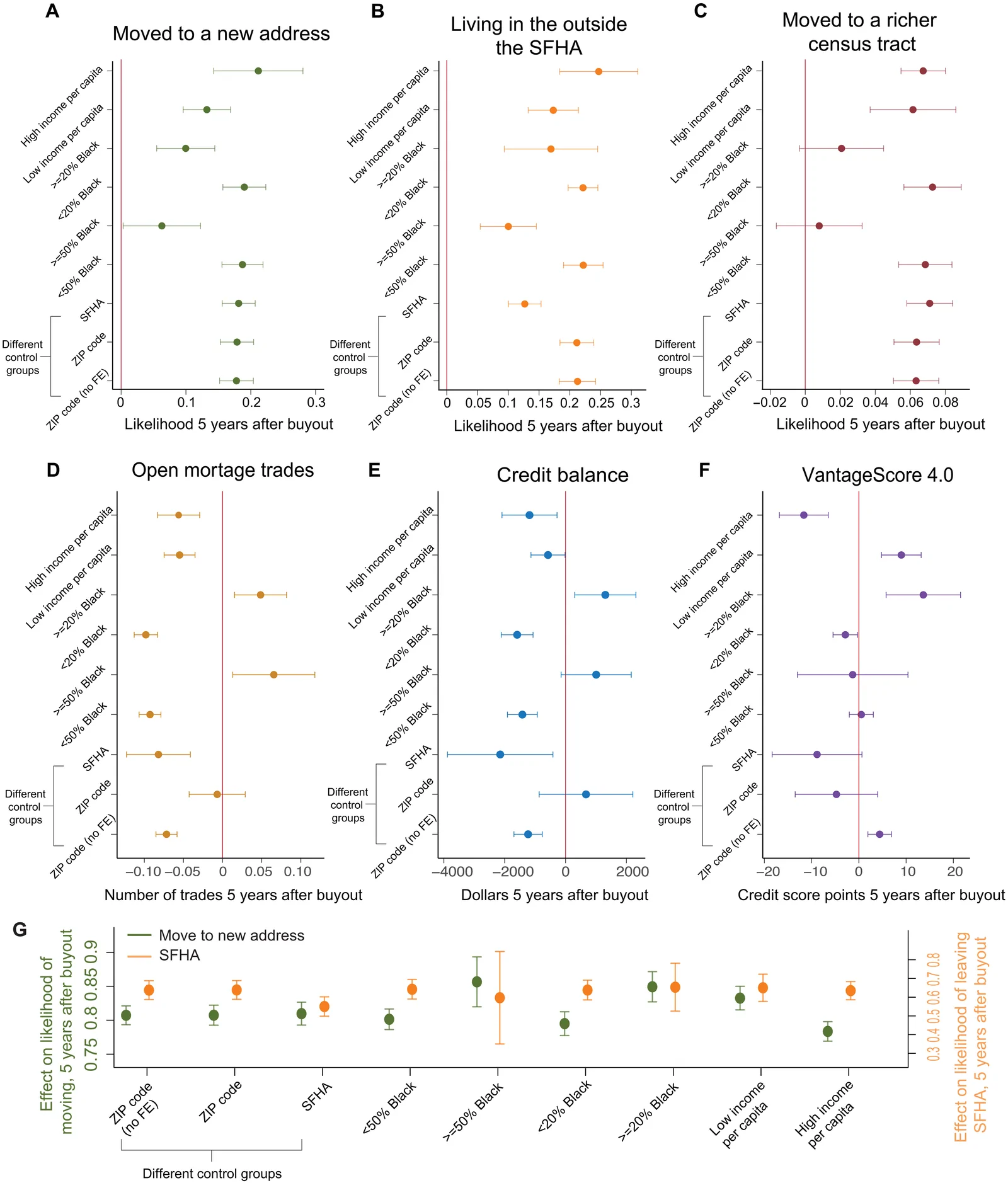
Differential effects of the buyout across race and income. Estimated cumulative effects 5 years after the buyout, relative to three versions of the control group: (i) all individuals living in buyout ZIP codes at the time of the buyout [“ZIP code (no FE)”]; (ii) individuals in the buyout ZIP code within the same state-cohort (“ZIP code”); (iii) individuals in the buyout ZIP code and SFHA within the same state-cohort (“SFHA”). Heterogeneous effects compare individuals living in high-fraction Black (≥50% Black and ≥20% Black) versus low-fraction Black (<50% Black and <20% Black) census tracts and high-income versus low-income per-capita census tracts. Error bars show 95% confidence intervals. (**A**) Moving to a new address. (**B**) Residing outside the SFHA. (**C**) Relocating to a higher-income census tract. (**D**) Number of open mortgage accounts. (**E**) Credit card balance (USD). (**F**) Credit score (VantageScore 4.0). (**G**) Cumulative effect on moving to a new address and on moving out of the SFHA, restricting the treated group to movers.

## RESULTS

### Buyouts increase and accelerate relocation

Residential relocation is a primary mechanism through which buyout programs may reduce household exposure to flood risk. Consistent with this objective, buyout participants relocate at substantially higher rates than comparable households following program approval (Fig. 2A). In the first 5 years after approval, participants are about 20 ± 1.1 percentage points (P<0.01) more likely to relocate than comparable households, and the effect remains 10 to 13 points higher even 15 years later. Conditional on movers, relocation rates rise by roughly 85 points relative to the control group (Fig. 3G). Because moving is itself affected by the program, these estimates should be interpreted as descriptive evidence on relocation destinations among households that ultimately moved. Similar increases in mobility are observed when restricting comparisons to households facing comparable baseline flood risks. Effects are generally the largest in riverine 1 and 0.2% annual-chance floodplains and remain positive across higher-risk flood categories (figs. S4 and S8). Together, the results suggest that buyouts substantially accelerate relocation from high-risk areas relative to the gradual mobility observed among untreated households while also contributing to additional long-run relocation beyond baseline trends.

Most moves are short-distance: Half of participants resettle within 10 km of their original homes (Fig. 1C). Compared with controls, buyout recipients are about 14 ± 1.1 points (P<0.01) more likely to live in a new ZIP code but not more likely to live in a different state (Fig. 2B and fig. S9). Thus, managed retreat under the program is largely local. The program reshapes neighborhood housing markets rather than producing large-scale out-migration. While such shifts can modestly erode local tax bases, they have limited implications for state revenues (*20*–*22*).

### Large near-term reductions in SFHA residence

Buyout participants frequently relocate away from high flood-risk zones. Within 5 years, about 65% of movers leave FEMA-designated SFHAs, most resettling in adjacent lower-risk zones (Fig. 1D and fig. S10). Relative to comparable households, the program increases the likelihood of residing outside the SFHA by roughly 13 ± 1.4 percentage points (P<0.01) shortly after approval, although the gap narrows to about 5 points after 15 years as untreated households also gradually relocate over time (Fig. 2C). The estimated reductions in residence within SFHAs are not confined to a single flood-risk subgroup. Positive effects are observed across most higher-risk flood categories, particularly among households initially residing in riverine 1% annual-chance floodplains and 0.2% annual-chance floodplains (fig. S11). Fewer than 1 in 10 buyout participants relocate to another SFHA property. In complementary analyses restricted to households that ultimately relocate, movement to lower-risk areas rises by roughly 55 points (P<0.0001) (Fig. 3G and fig. S8C). Together, these results suggest that FEMA buyouts substantially reduce direct flood exposure in the near term, even as longer-run residential mobility gradually reduces differences between treated and untreated households.

### Participants relocate to more advantaged areas

Place matters for long-term outcomes, and buyouts may allow households to relocate to areas with stronger economic prospects (*23*–*25*). To test this, I compare the census-tract characteristics of participants’ origin and destination addresses with those of similar households using prebuyout data from the 2000 Decennial Census to avoid contamination by postprogram changes.

Five years after approval, buyout participants are about 7.2 (±0.8, P<0.001) percentage points more likely than the controls to live in tracts with higher per-capita income, 7.3 (±0.9, P<0.001) points more likely to live in tracts with higher employment rates, and an 8.3 (±1.4, P<0.001) points more likely to live in tracts with greater college attainment (Fig. 2D). These results indicate that participants tend to move to nearby but more economically advantaged neighborhoods. This effect is robust when comparing individuals living in the same baseline flood-risk group before the buyout (fig. S12). Conditional on buyout movers, relocation to more economically advantageous neighborhoods is larger than the corresponding unconditional average treatment effect (fig. S8D). The estimated effects are also robust to specifications that control for pretreatment mobility propensity and to analyses restricted to households observed relocating during the posttreatment period (fig. S13).

### Short-term debt reductions and no lasting credit impacts

Buyout participation temporarily alters household borrowing patterns but does not meaningfully affect long-term credit access or creditworthiness. Before the program, participants held an average of 0.49 ± 0.74 open mortgages (tables S2 and S3). Four years after approval, they have 0.07 ± 0.015 fewer mortgages than comparable households (P<0.001; Fig. 2E), a decline that persists for roughly 7 years before converging with controls—consistent with participants repurchasing homes several years after relocation.

Participants also experience a short-run decline in credit-card balances, with average reductions of about $1694 ± 710 (P<0.05) 3 years after the program and $1069 ± 733 (P=0.14) after 5 years (Fig. 2F). The effect dissipates within a decade and is stronger among movers and in higher-income or predominantly white origin neighborhoods (Fig. 3E). Available credit and nonrevolving debts (auto and student loans) show no substantial change (Fig. 2H and fig. S14). These patterns suggest that some households use buyout proceeds to pay down debt (Fig. 1E) but that the liquidity effect is short-lived.

Contrary to concerns that long processing times might harm financial standing, credit risk scores remain stable or slightly improve. Mean VantageScore 4.0 credit scores rise marginally, from 713 to 718 for movers and 672 to 681 for nonmovers, with no statistically significant difference from controls (Figs. 1F and 2G and table S2). Overall, the buyout program appears to leave credit access and creditworthiness unaffected while providing a brief reduction in debt burdens during relocation.

### Larger relocation responses in higher-income areas

Program impacts differ modestly by neighborhood income. Contrary to expectations that wealthier households would rely less on assistance, buyouts have a somewhat stronger effect in higher-income tracts: Residents there are about 21.1 ± 3.5 percentage points (P<0.01) more likely to relocate after approval, compared with 13.2 ± 1.9 points (P<0.01) in lower-income tracts (Fig. 3A). High-income participants are also more likely to move to new ZIP codes (fig. S9), although both income groups show similar gains in neighborhood quality. Five years after the program, participants from high- and low-income areas are roughly 6 or 7 points more likely than controls to live in tracts with higher per-capita income, employment, and education (Fig. 3C).

Credit effects differ slightly by income. While mortgage counts and credit-card balances do not vary significantly across groups, participants from lower-income tracts experience small but significant improvements in credit risk scores (+9 ± 4 points, P<0.05; Fig. 3F and fig. S15). This suggests that the program modestly strengthens financial standing for lower-income participants who may otherwise struggle to rebuild credit after disasters.

### Smaller risk reduction in majority-Black areas

Neighborhood racial composition strongly shapes program outcomes. Residents of tracts with larger Black populations are markedly less likely to relocate after approval. In tracts with ≥50% Black population, the relocation effect is 6.3 ± 3.0 (P=0.044), roughly one-third of that observed in tracts with <20% Black residents (18.1 ± 1.3, P<0.001; Fig. 3A). Participants from higher-fraction Black tracts also move more slowly, with relocation rates resembling those of controls for several years postbuyout (fig. S7). Yet, among those who eventually move, destinations are comparable: Movers from high-fraction Black tracts are just as likely to relocate to lower flood-risk areas as their counterparts from <20% Black census tracts (Fig. 3G).

Differences persist in risk reduction and financial outcomes. Households from majority-Black tracts are less likely to move out of flood zones (6.3 ± 3.0, P=0.044, Fig. 3B) and see smaller gains in neighborhood income (3 or 4 versus 8 points; Fig. 3C). Consistent with these patterns, I find no measurable improvement in credit outcomes for high-fraction Black tracts, while residents from lower-fraction tracts experience declines in mortgage counts (about 0.1, P<0.001) and credit-card debt (about $1500, P<0.05), consistent with debt repayment (Fig. 3, D and E, and fig. S16).

Together, these results suggest inequities in benefits from the buyout program. Households in lower-income or majority-Black neighborhoods experience slower transitions out of SFHA areas and weaker financial gains, underscoring the need for more inclusive and timely implementation.

## DISCUSSION

### Flood-risk reduction and program performance

The FEMA property-buyout program increases the likelihood of residing outside FEMA-designated flood hazard areas, although a minority of participants relocate to properties that remain within SFHAs. Comparing buyout recipients with matched households in nearby flood-prone areas shows that recipients are significantly more likely to relocate to lower-risk zones, particularly those who were inside FEMA SFHAs before the program (fig. S3).

Reductions in flood exposure are evident across multiple baseline flood-risk environments and are the largest among households residing in riverine 1% annual-chance floodplains and 0.2% annual-chance floodplains before buyout approval (fig. S11). This pattern suggests that program-induced SFHA exit is the largest among households initially residing in higher-risk flood categories, consistent with the targeting of buyouts toward repeatedly flooded properties. Although the comparison groups account for differences in baseline flood risk, they cannot account for differences in realized damage from the flood events preceding buyout participation. More severely affected households may be more likely to participate in a buyout and, independently, may update their perceptions of future flood risk more strongly and prefer lower-risk destinations. Differences in realized flood damage may therefore contribute to the observed differences in postbuyout relocation and flood exposure.

Despite this progress, a minority of participants relocate to new properties that remain within SFHAs, meaning that the program does not eliminate risk entirely. As development expands and climate change amplifies coastal flooding, even currently low-risk destinations may face increasing hazard exposure (*26*–*28*). Strengthening postbuyout guidance and ensuring that updated flood maps inform relocation choices could enhance the program’s long-term effectiveness.

### Equity in access and outcomes

The buyout program’s benefits are not evenly distributed. Participants from higher-income neighborhoods are more likely to relocate and to move to areas with higher income, education, and employment opportunities, while households from majority-Black or low-income neighborhoods move less frequently and experience smaller improvements in neighborhood quality (Fig. 3). These tract-level heterogeneity estimates likely reflect broader neighborhood socioeconomic differences correlated with racial composition, housing values, and income, rather than causal effects of individual income or race alone.

#### Difference across neighborhood income levels

The results across income groups reveal a more nuanced pattern. Consistent with expectations, individuals in the control group originating from higher-income census tracts are more likely to move than those from lower-income census tracts (fig. S7, A and B), suggesting greater baseline mobility in higher-income areas. One might therefore expect the buyout program to have a larger effect among households from lower-income neighborhoods, where financial constraints could limit relocation absent program assistance. However, I find that the estimated effect of the buyout on moving is slightly larger for individuals from higher-income census tracts, although the difference is not statistically significant. In contrast, among treated households that moved following a buyout, those originating from lower-income neighborhoods are more likely to relocate to higher-income neighborhoods than their counterparts from higher-income areas (fig. S8). Together, these findings suggest that while the program does not differentially increase mobility across income groups, it may facilitate access to higher-income destination neighborhoods for households originating from lower-income areas.

#### Difference across neighborhood race composition

For participants originating from majority-Black census tracts, postbuyout relocation rates are lower than those from high-fraction white population areas. The likelihood of moving is lower for buyout participants in high-fraction Black census tracts, and it does not differ from the control group until 3 years postbuyout (fig. S7, C and D). Relocation rates remain persistently lower throughout the postbuyout period, suggesting a slower or more difficult postbuyout relocation process.

One possible explanation is that households originating from majority-Black census tracts face different housing-market constraints following buyout participation, including lower average buyout compensation and differences in local housing affordability. According to OpenFEMA data, the average buyout payment is $40,583 in high-fraction Black census tracts compared to $93,977 in high-fraction white tracts (*10*). This difference is consistent with prior work documenting racial inequities in housing markets, including lower average property values in many predominantly Black communities (*29*–*31*). Consequently, participants in these neighborhoods may face greater difficulty purchasing comparable homes outside high-risk flood areas using buyout compensation alone. The persistent decline in the number of mortgages for this group (Fig. 3D and fig. S16A) provides suggestive evidence consistent with greater difficulty purchasing homes following buyouts. In addition, while individuals in majority white neighborhoods experience temporary declines in their credit balances following a buyout, individuals in majority Black neighborhoods do not (Fig. 3E and fig. S16B), although estimates for the latter group are substantially less precise because of smaller sample sizes. These patterns suggest the importance of further research into racial disparities in postbuyout relocation constraints and housing market access.

### Fiscal implications and broader benefits

A back-of-the-envelope cost-benefit analysis (Fig. 4) shows that the fiscal efficiency of the buyout program depends critically on the extent to which the program induces sustained reductions in flood-risk exposure. I evaluate three scenarios on the basis of the empirical results of the analysis (Fig. 4A):

1) Full program-induced relocation (baseline), in which all observed relocation among buyout recipients is attributed to the program and households relocate to lower-risk areas.

2) Estimated move effect, which scales benefits using the estimated cumulative treatment effect on moving.

3) Estimated SFHA exit effect (preferred), which scales benefits using the estimated cumulative treatment effect on no longer residing in an SFHA, allowing movers to relocate either to lower-risk areas or to other flood-prone locations.

**Fig. 4.**
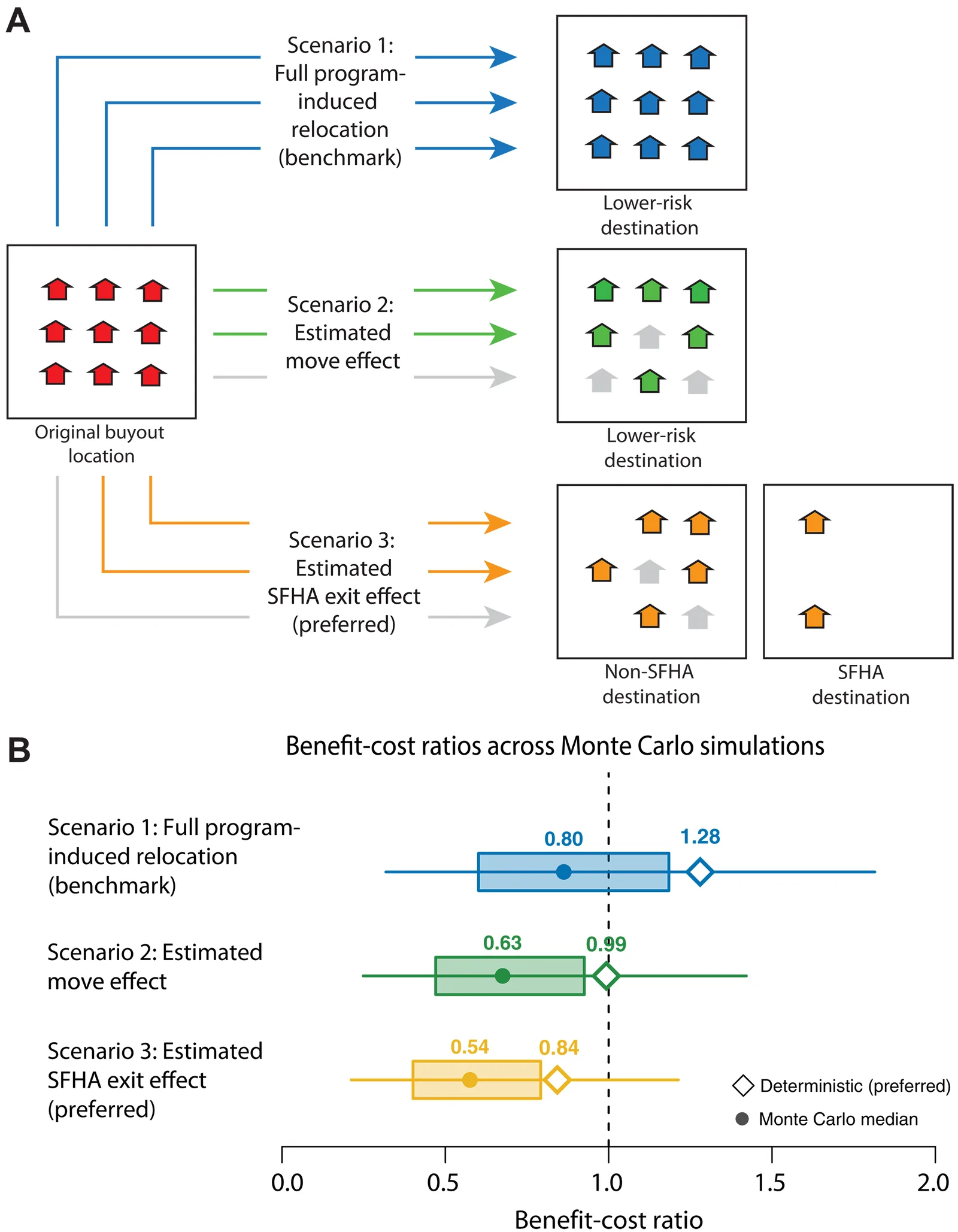
Benefit-cost analysis of the buyout program. (**A**) Illustration of the three alternative assumptions regarding realized reductions in flood-risk exposure. Scenario 1 (“Full program-induced relocation”) attributes all observed relocation to buyout participation. Scenario 2 (“Estimated move effect”) scales benefits using the estimated cumulative treatment effect on residential mobility. Scenario 3 (“Estimated SFHA exit effect”), the preferred specification, scales benefits using the estimated cumulative treatment effect on no longer residing in an SFHA, allowing incomplete reductions in realized flood-risk exposure. Gray arrows and houses indicate background mobility not attributed to buyout participation. (**B**) Monte Carlo distributions of benefit-cost ratios under each scenario, with deterministic point estimates under preferred assumptions shown for comparison. Boxes represent interquartile ranges, whiskers represent 5th to 95th percentile intervals, filled points denote median simulated benefit-cost ratios, and open diamonds denote deterministic benefit-cost ratios. Simulations incorporate uncertainty in treatment effects, acquisition and implementation costs, discount rates, disaster assistance expenditures, and other model assumptions. The dashed vertical line indicates a benefit-cost ratio of 1.

These scenarios progressively relax strong assumptions regarding relocation and realized reductions in flood-risk exposure. Scenario 1 therefore serves as an upper-bound benchmark, while scenario 3 provides the preferred and most conservative specification because it directly incorporates the counterfactual relocation out of high-flood-risk areas, as observed in the empirical analysis (Figs. 2C and 3, B and G).

The analysis incorporates uncertainty in treatment effects, acquisition and implementation costs, discount rates, and conditional building and content losses using Monte Carlo simulations and sensitivity analyses (Fig. 4B). Because the recurrence of damaging floods among acquired properties is not directly observed, the results are evaluated separately across three recurrence benchmarks. Under scenario 1 (“Full program-induced relocation”), the benefit-cost ratio is 1.28 under deterministic point-estimate assumptions (median of 0.80 across Monte Carlo simulations), declining to 0.99 (median of 0.63) under scenario 2 (“Estimated move effect”) and 0.84 (median of 0.54) under scenario 3 (“Estimated SFHA exit effect”), with 32.7, 17.3, and 9.9% of simulations exceeding a benefit-cost ratio of 1, respectively (Fig. 4B and fig. S17). The deterministic estimates are calculated at the 30-year horizon and 3% discount rate, with alternative 10- and 20-year horizons reported as sensitivity analyses (fig. S18).

This decline in the ratios reflects incomplete realized reductions in flood-risk exposure, background mobility among untreated households, implementation costs, and convergence in relocation patterns over time. Shorter benefit horizons and higher discount rates substantially reduce estimated benefit-cost ratios (fig. S18), particularly in the estimated move-effect and SFHA-exit scenarios. Sensitivity analyses further show that the results are most driven by uncertainty regarding acquisition costs, damaging-flood recurrence, conditional building and content loss, and the persistence of realized reductions in flood-risk exposure, highlighting the importance of sustained reductions in exposure and effective targeting of high-risk properties (fig. S19). We additionally report benefit-cost ratios across the full range of recurrence benchmarks to show how the estimated ratio varies with the assumed frequency of damaging floods (fig. S20). The recurrence probability required for benefits to equal costs (BCR = 1) ranges from 15.5% under full relocation to 20.2% under the estimated move effect and 23.9% under the estimated SFHA-exit effect, the latter exceeding even the upper recurrence benchmark (23.3%) (fig. S20).

Because the analysis focuses primarily on avoided building and content losses while excluding many broader social and environmental benefits, these estimates should be interpreted as conservative estimates of the benefits of the program rather than comprehensive measures of social welfare. Additional gains—such as improved physical and mental health from reduced disaster exposure (*23*, *32*), spillover reductions in neighboring flood damages (*33*, *34*), and ecosystem services from restored wetlands (*35*)—could further increase the broader social value of buyout programs. Together, these results suggest that the long-run value of buyout programs depends not only on the benefits generated by relocation itself but also on how efficiently those benefits are delivered. While the program substantially increases the likelihood of residing outside FEMA-designated flood hazard areas relative to comparable untreated households, benefit-cost performance is highly sensitive to implementation delays, incomplete relocation, and the extent to which households would have relocated even in the absence of participation.

## MATERIALS AND METHODS

### Human subject approval

This study was approved by the Princeton University Institutional Review Board (IRB no. 14245; approved 17 January 2023). Informed consent was waived because the research involves only secondary analysis of deidentified administrative and commercial data.

### Data sources

#### Infutor individual address data

To measure individual locations and moves, I use data from Lead Intelligence dba InfutorData (Infutor), a private consumer reference data company. Infutor aggregates over 560 million records, spanning a panel of more than 260 million individual demographic profiles and addresses from the 1980s through August 2020 (although data completeness for the US population begins in 2000). The data contain street addresses and the date of residence, first and last name, demographic information, and first five digits of the individual’s social security number. Demographic information covers gender, year and month of birth, and year and month of death where applicable. Information such as race, employment, income, and education are not available.

Infutor identifies and authenticates individual addresses using numerous sources such as the National Change of Address, voter registration, deed, property data, automotive sales phonebooks, magazine subscriptions, real estate data, and credit header files. Each address undergoes authentication from at least two direct sources before being recorded in the individual data profiles.

#### Census tract characteristics

I assembled census tract characteristics for the treated and control addresses for data validation, heterogeneity analysis, and estimating effects on neighborhood characteristics. These census tract characteristics are from the US Decennial Census (2000, 2010, and 2020) (*36*–*38*).

#### Credit report data

I use individual-level credit and debt data from one of the major credit bureaus to evaluate changes in household finances. The data provider is one of the largest consumer credit repositories and credit scoring companies in the US and collects comprehensive information about individual credit histories, including the number of loans, frequency of payments, and amounts owed. The consumer credit account information includes home loans, auto loans, credit card accounts, and student loans. Credit card accounts cover all types of credit issuers, including banks, bank card companies, credit unions, savings and loan associations, and department stores and other retailers. The credit bureaus also provide the VantageScore 4.0 credit score, a trademarked measure of consumer credit risk ranging from 300 to 850. Higher scores indicate higher creditworthiness, with a score of 697 considered “prime,” one tier below the best range (i.e., a good score). The VantageScore 4.0 credit score is developed by the credit bureaus in collaboration with other credit bureaus.

#### FEMA buyout program data

Buyout program participants are identified using data sourced from the National Public Radio (NPR) under their 2017 Freedom of Information Act request. This dataset includes names, addresses, fiscal years (corresponding to the year of the disaster or funding appropriation), and purchase prices for houses acquired through FEMA property acquisition programs spanning 1989 to 2017. It encompasses acquisitions under FEMA’s major programs including the Hazard Mitigation Grant Program, Flood Mitigation Assistance, and Pre-Disaster Mitigation (now called the Building Resilient Infrastructure and Communities Grant Program) (table S1).

#### FEMA flood maps

Property-level flood risk is assessed using FEMA’s National Flood Hazard Layer flood hazard maps, which dictate participation in the National Flood Insurance Program (NFIP) and provide comprehensive flood risk data across the United States. The Flood Insurance Rate Map and Letters of Map Change are components of these maps, encompassing Geographic Information System boundary layers of flood zones, base flood elevation, and floodway status for specific locations. Key risk classifications include the 1-percent-annual-chance flood event, the 0.2-percent-annual-chance flood event, and areas of minimal flood risk. Properties within the 1-percent-annual-chance flood event boundary, known as SFHAs, are identified. I use these layers to determine whether properties in both treated and control groups fall within SFHAs, as well as to assess flood risk for properties individuals move to. This serves as a proxy for flood risk and provides property owners with clear notification of their participation requirement in NFIP.

#### Additional FEMA program data

I quantify additional costs associated with the FEMA buyout program and flood losses using FEMA administrative data and NFIP claims from the OpenFEMA database. The NFIP data provide individual-level flood insurance claims, ensuring privacy by anonymizing data up to the census tract level, reported ZIP codes, and precise latitude and longitude to one decimal point (*10*). The dataset covers the entire history of program claims from 1978 to the present day. Detailed information on building and content claims payments and characteristics of the flood loss and insured property are included. While the data record the year of the flood loss event, they do not include the date of fund disbursement to the household. Policy and claims data are anonymized to the census tract, reported ZIP code, and one decimal point digit of latitude and longitude.

I use these claims data to characterize building and content losses associated with damaging floods for properties in areas represented in the buyout sample. For the benefit-cost analysis, I restrict claims to properties in FEMA A and V flood zones located in ZIP codes represented in the buyout sample and sum building and content payments within each paid claim. These data are used to estimate mean building and content losses conditional on a paid flood claim, which are then combined with alternative benchmarks for the recurrence of damaging floods to estimate expected annual avoided damages.

I also use the OpenFEMA datasets (*10*) for FEMA’s Individual Assistance (IA) programs, including the Individuals and Households Program (IHP), which comprises Housing Assistance and Other Needs Assistance. The dataset includes information on the number of grant applicants, year, county, ZIP code, inspections, severity of damage, assistance provided, owner or renter status, and grant amounts for each applicant. These data form the basis for estimating avoided disaster-assistance expenditures per household in the benefit-cost analysis.

### Data processing and sample construction

#### Constructing individual address histories

Infutor assigns an effective date to each address, representing the date when they confirm the individual’s association with that address. I make corrections for the last date in the panel on the basis of an individual’s date of death (if relevant). Nonresidential addresses, such as P.O. boxes, are omitted if they appear with the same effective date as another residential address for the same individual.

#### Measuring moves

To measure moves using the Infutor data, I construct a year-over-year migration variable on the basis of whether the individual is observed at a new address within a given year. For example, if I observe a person *i* with two addresses ($A_{i}^{1}$ and $A_{i}^{2}$), each with an associated effective year, $E_{i}^{1}$ and $E_{i}^{2}$, respectively, where $E_{i}^{1}<E_{i}^{2}$, I assume that individual *i* resides at address $A_{i}^{1}$ from $E_{i}^{1}$ to $E_{i}^{2}-1$ and at address $A_{i}^{2}$ from $E_{i}^{2}$ onward. Consequently, I infer that the individual moved in year $E_{i}^{2}$. This produces a consistent year-over-year migration history for each individual.

In instances where Infutor reports multiple addresses with the same effective year, I determine the correct ordering using the dataset’s supplemental timing fields—specifically, the effective-date correction variable, the calendar month (when available), and Infutor’s internal rank ordering of addresses within a year. These tie-breaking rules ensure a unique and temporally consistent address sequence before constructing the migration indicator.

#### Matching FEMA buyout addresses to Infutor

To identify buyout participants in the Infutor individual address data, I matched the buyout addresses with the addresses in the Infutor data. The total number of buyouts in my sample from 1989 to 2017 is 41,004 properties, and from 2000 to 2017, it is 19,169 properties. I performed the matching on the full sample of buyouts between 1989 and 2017, but because the Infutor data are limited in the 1990s, I restricted my treatment to buyouts that occur in 2000 to 2017. I conducted robustness checks on subsets from 2010 to 2017, and the results are consistent with the main findings. I used three separate matching procedures:

1) Exact match on address, surname, and year of the buyout.

2) Fuzzy match on address and surname.

3) Exact match on cleaned address and buyout year.

#### Exact match on address and surname

This method results in many unmatched properties because only 43% of NPR addresses include an owner name, and these names contain errors requiring cleaning. It also restricts matches to individuals sharing the surname of the recorded owner—missing renters, cohabitants, and other adults. The match rate is 9% for both 1990 to 2017 and 2000 to 2017.

#### Probabilistic matching (fastLink)

Using Levenshtein distance with a threshold of 0.93, I identified 7631 people with matched buyout addresses. Of these, 5862 moved to new addresses and 3848 moved twice, totaling 24,148 addresses. Manual auditing revealed many false positives, even with conservative thresholds, leading me to exclude this method from the final dataset.

#### Exact match on cleaned address and buyout year (preferred)

After cleaning and standardizing addresses, I conducted exact matching on full address fields, ensuring that the individual lived at the buyout address during the buyout year (either disaster year or fiscal year when funding occurred). This approach yields a 47.5% match rate for 2000 to 2017, peaking at 80% in 2016 (fig. S21). I identified 14,999 participants across the US, averaging 833 treated individuals per cohort year. Unmatched addresses often correspond to entire buyout projects that never appear in Infutor, likely because residents moved before Infutor captured their residency. These missingness patterns are absorbed by fixed effects (fig. S22).

Match rates also vary across states. States with very few participants often show either very high or very low match rates depending on the timing of their buyouts. For example, Idaho has only 19 buyout properties—all occurring after 2010—and a 100% match rate. Utah, by contrast, has 87 buyouts but a much lower match rate (2%), primarily because its buyouts occurred earlier (pre-2010), when individuals were less likely to appear in the Infutor sample.

#### Constructing the control groups

Because participation in the buyout program is voluntary, the ideal counterfactual is the treated individual in a world where they did not receive a buyout (*39*). As this cannot be observed, I construct two control groups using Infutor: (i) individuals living in ZIP codes where buyouts occur in the years of buyout programs; (ii) the subset of these individuals who also reside in SFHAs.

To implement these comparisons, I identify ZIP codes with buyouts in each year and then draw individuals living in those ZIP codes during that year. I match their addresses to FEMA flood hazard layers to determine SFHA status. The ZIP-only group includes 705,874 individuals; the SFHA-restricted group includes 47,973 individuals.

To further characterize underlying flood exposure, I combine detailed FEMA flood zone classifications into broader flood-risk categories on the basis of flood probability and flood type. Specifically, coastal V/VE zones are grouped as “coastal 1% flood risk,” inland A/AE/AH/AO zones as “riverine 1% flood risk,” 0.2% annual-chance floodplains as a separate category, and areas outside mapped flood hazard zones as “minimal flood risk.” Flood-risk categories are fixed at each property’s event year (τ = 0) and then held constant over time to avoid changes in FEMA map designations mechanically affecting comparisons.

The composition of the treated and untreated samples differs substantially in the full ZIP-level sample. Untreated observations are disproportionately concentrated in minimal-risk areas, whereas treated observations are primarily located in higher-risk flood zones, particularly riverine 1% floodplains. As a result, the unrestricted ZIP-level comparison implicitly contrasts high-risk treated properties with predominantly low-risk controls, generating less comparable pretreatment trends. Restricting the comparison group to SFHA areas substantially improves balance in baseline flood exposure and yields the closest pretreatment alignment in the event-study analyses (tables S2 and S3 and fig. S13). For these reasons, the SFHA-restricted sample is the preferred control group.

In addition to these primary comparison groups, I estimate two alternative specifications to assess the sensitivity of the results to differences in baseline mobility behavior. First, I reestimate the event-study models while flexibly controlling for individual-level pretreatment mobility propensity using observed address changes before buyout participation (fig. S13). Second, I estimate specifications that restrict the comparison to households that eventually relocate during the posttreatment period (fig. S13). These alternative specifications do not define new primary control groups but instead provide complementary ways of constructing the counterfactual and assessing whether the estimated effects are sensitive to differences in underlying mobility patterns between treated and untreated households.

Last, because buyout participation substantially increases relocation, I estimate complementary mover-conditioned specifications that restrict the sample to households observed relocating during the posttreatment period. These analyses help distinguish whether reductions in residence within FEMA-designated flood hazard areas arise primarily from differences in the likelihood of moving or from differences in relocation destinations conditional on moving.

### Identification strategy and estimation

#### Conceptual identification

The goal of the analysis is to estimate the causal effect of receiving a buyout on relocation and financial patterns. The primary challenge is that participation in the buyout program is voluntary and, therefore, not randomly assigned. Previous studies have identified distinct demographic differences between buyout participants and their nonparticipating neighbors (*5*, *6*). For example, buyout participants tend to reside in census tracts with higher median incomes, greater educational attainment, and larger proportions of white residents compared to nonparticipants in the same ZIP codes (tables S2 and S3). Therefore, these groups may have exhibited a predisposition toward moving even in the absence of the buyout, potentially to more desirable locations, or they may have a stronger inclination toward relocation.

Because the buyout process is often lengthy and involves multiple stages between the initial disaster event, application, approval, and final property acquisition, the main estimates are best interpreted as intent-to-treat effects of assignment to and participation in the buyout process. These estimates are therefore inherently conservative with respect to realized reductions in flood-risk exposure, but they highlight important issues related to implementation delays, incomplete relocation, and the nonrandomness of program participation.

Complementary analyses restrict attention to households that ultimately relocate following approval. Because posttreatment mobility is itself affected by program participation, these estimates are interpreted as descriptive evidence on relocation destinations conditional on moving rather than as a separate causal estimand.

#### Difference-in-differences model

To estimate the difference in means, or the intent to treat, for the outcomes of interest, I use linear regression. I run eight versions of the model with different location and time interacted fixed effects. I do not find statistically significant differences in the effect on moving between the different versions. Table S5 shows the main effect of moving to a different address for treated individuals 5 years after the buyout across the four models. Therefore, Eq. 1 is my preferred specification.

$$y_{\mathrm{it}}=\sum_{\tau =-10}^{15}\beta_{\tau }\cdot 1\left[\text{buyou}t_{i\tau }\right]+\mu_{s\times m}+\gamma_{c}+\delta_{t}+\epsilon_{\mathrm{it}}$$ (1)

where $y_{\mathrm{it}}$ denotes the outcome for individual *i* in year *t*, and $1\left[\text{buyou}t_{i\tau }\right]$ is an indicator equal to 1 if individual *i* received a buyout τ years before or after year *t* ($\tau =t-m_{i}$). The coefficients $\beta_{\tau }$ trace the dynamic effects of buyout participation relative to the year before treatment. The model includes state-by-cohort fixed effects ($\mu_{s\times m}$), county fixed effects ($\gamma_{c}$), and year fixed effects ($\delta_{t}$). Standard errors are clustered at the state level.

Primary outcomes include indicators for whether an individual (i) remains at their buyout address, (ii) lives within the same ZIP code or state, (iii) resides in an SFHA, or (iv) lives in a census tract with above-median household income (2000 Census baseline). To summarize the total effect over time, I also estimate a cumulative version of the event-study model. Let ${\hat{\beta }}_{\tau }$ denote the estimated coefficient at event time τ. The cumulative treatment effect through horizon *k* is defined as

$$\Gamma_{k}=\sum_{\tau =0}^{k}{\hat{\beta }}_{\tau }$$ (2)

where $\Gamma_{k}$ measures the cumulative change in the probability of moving from the year of buyout through *k* years after treatment, relative to the pretreatment baseline. This provides an interpretable measure of the total effect of the program over time rather than the effect in any single year. This is the main result displayed in Fig. 3.

Recognizing that full program participation ultimately requires households to vacate acquired properties, the main estimates are best interpreted as intent-to-treat effects of assignment to and participation in the buyout process. I also report complementary estimates restricted to treated households that relocated at some point after *t* = 0 using the same regression framework but defining treatment only among posttreatment movers. Because posttreatment mobility is itself an outcome of the program, these estimates should be interpreted as descriptive evidence on outcomes among households that ultimately relocated following buyout participation rather than as a separate causal estimand.

#### Contemporaneous flood damage and interpretation

An additional identification concern is selection based on the severity of the realized flood event. Even within the same ex ante flood-risk category, buyout participants may have experienced more severe realized damage than comparison households. More severely damaged households may be more likely to participate because the relative value of a buyout increases with damage severity, and damage itself may increase the likelihood of relocation independent of the program. More severe damage may also lead households to update their perceptions of future flood risk more strongly than less-affected households, potentially increasing their likelihood of choosing lower-risk destinations even in the absence of a buyout. Because these damage shocks occur contemporaneously with the buyout process, flat pretreatment trends cannot rule out this source of selection. Restricting comparisons to households within SFHAs and conducting complementary analyses within finer flood-risk categories improve comparability in underlying flood-hazard exposure but cannot eliminate potential selection on the basis of realized damage severity.

### Robustness and alternative specifications

#### Comparison groups

I reestimate the event-study models using both control groups: (i) individuals in ZIP codes where a buyout occurred in the cohort year and (ii) those individuals residing in SFHAs. The SFHA-restricted group exhibits stronger pretreatment alignment with the treated group and is therefore used in the main results. ZIP-only estimates are shown for comparison (Fig. 3, table S5, and fig. S3).

#### Flood-risk subgroups

To assess whether differences in baseline flood exposure drive the full-sample pretreatment trends, I reestimate the event-study models separately by baseline flood-risk category using flood-risk classifications fixed at the property’s event year. The subgroup analyses show that postbuyout increases in moving occur across high-risk flood categories, particularly riverine 1 and 0.2% annual-chance floodplains, while exhibiting substantially flatter pretreatment trends than the unrestricted sample. In contrast, the minimal-risk subgroup exhibits noisier pretreatment dynamics, consistent with greater selection into program participation outside mapped high-risk flood zones. These results suggest that the primary findings are not driven by comparisons between high-risk treated properties and low-risk controls and that the estimated mobility effects are broadly consistent across high-risk flood environments (figs. S4 and S11).

#### Conditional-on-moving comparisons

Because buyout participants relocate at substantially higher rates than untreated households, the main estimates combine two margins: whether households move and where they relocate conditional on moving. To separate these mechanisms, I estimate complementary event-study models restricted to households observed relocating during the posttreatment period in both the treated and control groups. The estimating equation is identical to the primary specification (Eq. 1), but the sample is restricted to households observed relocating during the posttreatment period. These mover-conditioned specifications continue to show increases in residence outside SFHAs among buyout participants, particularly within higher-risk flood categories such as riverine 1% annual-chance floodplains and floodways (fig. S13). However, the mover-conditioned sample exhibits noisier and less stable pretreatment dynamics, reflecting the selected nature of households that relocate absent program participation. I therefore interpret these estimates as complementary to, rather than replacements for, the primary intent-to-treat style specifications.

#### Baseline mobility adjustment

To assess whether the estimated treatment effects are driven by preexisting differences in residential mobility propensity, I reestimate the event-study models while flexibly adjusting for baseline mobility behavior. Specifically, I construct an individual-level pretreatment move rate using observed address changes before buyout and interact this measure with event time indicators.

$$\begin{array}{c} y_{\mathrm{it}}=\sum_{\tau =-10}^{15}\beta_{\tau }\cdot 1\left[\text{buyou}t_{i\tau }\right]+\sum_{\tau =-10}^{15}\theta_{\tau }\cdot 1\left[\text{EventTim}e_{i\tau }\right]\times \text{PreMoveRat}e_{i}\\ +\mu_{s\times m}+\gamma_{c}+\delta_{t}+\epsilon_{\mathrm{it}} \end{array}$$ (3)

where ${\text{PreMoveRate}}_{i}$ is the individual-level pretreatment move rate, and the interaction with event-time indicators allows households with different baseline mobility propensities to follow distinct underlying mobility trajectories over time, rather than imposing parallel trends across individuals with substantially different pretreatment residential histories. The estimated postbuyout increases in residential mobility and reductions in residence within FEMA-designated flood hazard areas remain qualitatively similar under this adjustment, suggesting that the main findings are not driven solely by treated households having a greater underlying tendency to relocate before program participation (fig. S13).

#### Model variants

I estimate eight versions of the difference-in-differences model, adjusting location and time fixed effects. The estimated effects on moving are statistically indistinguishable across specifications. Table S5 summarizes the main effect at 5 years postbuyout.

#### Estimation of heterogeneous effects

To examine whether buyout responses vary across neighborhood contexts, I estimate heterogeneous treatment effects by census tract demographic and socioeconomic characteristics measured before buyout participation. Specifically, I classify individuals according to whether they resided in census tracts above or below the sample median for median household income and tract-level Black population share in the prebuyout year. These characteristics are fixed before treatment and therefore do not vary in response to buyout participation.

I estimate subgroup-specific event-study effects using a triple-difference specification that interacts treatment event-time indicators with indicators for the relevant tract-level subgroup

$$\begin{array}{c} y_{\mathrm{it}}=\sum_{\tau =-10}^{15}(\theta_{\tau }^{\text{low}\_\text{frac}\_\text{Black}}\cdot 1\left[\text{buyou}t_{i\tau }\right]\cdot Q_{i}^{\text{low}\_\text{frac}\_\text{Black}}\\ +\theta_{\tau }^{\text{high}\_\text{frac}\_\text{Black}}\cdot 1\left[\text{buyou}t_{i\tau }\right]\cdot Q_{i}^{\text{high}\_\text{frac}\_\text{Black}})\\ +\text{controls}+\epsilon_{\mathrm{it}} \end{array}$$ (4)

where $Q_{i}^{\text{low}\_\text{frac}\_\text{Black}}$ and $Q_{i}^{\text{high}\_\text{frac}\_\text{Black}}$ indicate the demographic categories of interest in the prebuyout year. The coefficients separately capture dynamic effects for each subgroup.

These estimates should be interpreted as heterogeneity across neighborhood environments rather than individual demographic characteristics. Because census tract racial composition, income, housing values, and other neighborhood characteristics are highly correlated, the results are not intended to isolate a causal effect of any single tract characteristic. Rather, they describe how buyout responses vary across different types of communities.

### Benefit-cost analysis

The benefit-cost analysis estimates the potential fiscal costs and avoided future public expenditures associated with FEMA buyout programs using empirically estimated treatment effects (Figs. 2 and 3), observed buyout expenditures (*40*), and historical NFIP and disaster assistance data (*41*) under three scenarios (Fig. 4A):

1) Full program-induced relocation (baseline), in which all observed relocation among buyout recipients is attributed to the program and all induced movers relocate to lower-risk areas.

2) Estimated move effect, which scales benefits using the estimated cumulative treatment effect on moving while assuming that induced movers relocate to lower-risk areas.

3) Estimated SFHA exit effect (preferred), which scales benefits using the estimated cumulative treatment effect on no longer residing in an SFHA, allowing some induced movers to relocate to other high-risk locations.

The three scenarios differ in the extent to which buyout participation is assumed to reduce realized future flood-risk exposure. The full relocation scenario assumes complete and permanent elimination of exposure among program-induced movers. In contrast, the estimated move-effect and SFHA-exit scenarios scale expected avoided future public expenditures using empirically estimated treatment effects on residential mobility and on no longer residing within an SFHA, respectively. Because some induced movers may relocate to other flood-prone locations, the preferred specifications do not assume universal or permanent elimination of flood exposure following buyout participation.

Benefits include avoided building and content losses, avoided IA expenditures, and an open-space amenity cobenefit associated with converting acquired properties to a permanent open space. Avoided building and content losses are estimated using historical NFIP claims. Losses conditional on a paid flood claim are estimated using NFIP claims for properties in FEMA A and V flood zones located in ZIP codes represented in the buyout sample (*10*). Building and content payments are summed within each paid claim, yielding a mean conditional loss of ∼$45,900 (range of $4911 to $55,629). In addition to avoided building content damage, the benefits account for avoided FEMA IHP disaster assistance, the primary form of federal individual assistance disbursed after a flood disaster declaration. As with the flood-damage term, this benefit accrues only in years a damaging flood occurs and is scaled by both the recurrence probability and scenario-specific risk-reduction effect using a mean per-property IHP payout of $4811 (range of $2131 to $9299 across years) (*41*). Both of these measures are scaled by flood recurrence probabilities. The benefit calculation also includes a fixed annual open-space amenity cobenefit of $250 per property (range of $0 to $2,000), which is not scaled by the recurrence probability or risk-reduction effect, given that it accrues regardless of whether a flood occurs in a given year.

Because the annual recurrence of damaging floods among acquired properties is not directly observed, I use a 20% annual recurrence benchmark, corresponding to two qualifying losses within 10 years and consistent with FEMA’s repetitive-loss classification. When adjusted, the preferred approximate mean annual building and content loss is $9180. I additionally evaluate sensitivity to the recurrence assumption using a lower benchmark of ∼1.5%, on the basis of observed NFIP paid-claim incidence among insured properties in comparable high-risk flood zones, and an upper benchmark of ∼23%, on the basis of the annual frequency of flood-related disaster declarations among counties represented in the buyout sample (figs. S19 and S20). These alternative values are intended to assess sensitivity to the recurrence assumption rather than to represent a statistical confidence interval. The annual benefit of a buyout in year *t* is calculated as

$$B_{t,s}=\left[{\hat{\text{RRE}}}_{s}\times p_{f}\times \left(\bar{D}+\bar{\text{IA}}\right)+\bar{A}\right]\times \phi \left(t\right)$$ (5)

where $\bar{D}$ is the mean building and content loss conditional on a paid NFIP claim, $\bar{\text{IA}}$ is the conditional IHP benefit per damaging flood event, and $p_{f}$ is the assumed annual recurrence probability of a damaging flood ($p_{f}\in \left\{1.5\%,20\%,\;23.3\%\right\}$). ${\hat{\text{RRE}}}_{s}$ is a scenario-specific risk-reduction effect representing the share of flood-related losses assumed to be avoided as a result of buyout participation, from ${\hat{\beta }}_{\tau }$ in Eq. 2. $\bar{A}$ is a fixed annual open-space amenity cobenefit that is not scaled by ${\hat{\text{RRE}}}_{s}$ or $p_{f}$, given that it accrues independent of whether a flood occurs.

Time variation in annual benefits enters through the phase-in factor ϕ(t), reflecting the time required for relocation, demolition, and site restoration to be completed. Benefits ramp from 5% of full value in year 1 to 100% by year 6 and are held constant thereafter. Benefits are evaluated over alternative 10-, 20-, and 30-year horizons. Discounted benefits over horizon *H* are

$${\text{PV}}_{s}=\sum_{t=1}^{H}\frac{B_{t,s}}{{\left(1+r\right)}^{t}}$$ (6)

where *r* is the discount rate. Uncertainty in ${\hat{\text{RRE}}}_{s}$ is propagated via Monte Carlo simulation, drawing the estimated risk-reduction effects from normal distributions defined by their respective point estimates and standard errors and truncating draws to [0,1]. The recurrence benchmarks are treated as sensitivity scenarios rather than sampled distributions and are held fixed within each Monte Carlo analysis. Uncertainty in program cost parameters is propagated jointly with uncertainty in the estimated risk-reduction effects.

Costs include observed acquisition expenditures reported in FEMA buyout data (*10*), together with additional implementation and transaction costs intended to capture activities such as project administration, planning, environmental review, legal/title work, and project management. Following work documenting substantial implementation costs in buyout programs (*19*), administrative and transaction costs are modeled as an additional share of direct program costs (acquisition, relocation assistance, and demolition/site restoration expenditures). The preferred specification uses the observed median acquisition cost of $87,160 per property, calculated from FEMA’s administrative data, together with separate assumptions for demolition and site restoration costs, implementation costs, and optional relocation assistance expenditures.

To characterize uncertainty, the analysis incorporates both Monte Carlo simulations and one-at-a-time sensitivity analyses over treatment effects, acquisition costs, implementation costs, conditional building and content losses, discount rates, and other assumptions. Because the recurrence of damaging floods among acquired properties is not directly observed, recurrence is evaluated separately using the three benchmarks described above rather than being assigned a probability distribution in the Monte Carlo simulations. The parameters jointly varied in the Monte Carlo simulations are the risk-reduction effect, conditional building and content loss, program cost parameters (acquisition, relocation, demolition, administrative/transaction, and deadweight costs), disaster assistance, the open-space amenity, the discount rate, and the horizon. The Monte Carlo simulations jointly vary the parameters using bounded probability distributions informed by FEMA administrative data (*41*) and the buyout literature (Fig. 4B and fig. S17, A and B) (*7*, *19*). Net present values are calculated using real discount rates (r) ranging from 1 to 7%, with 3% used as the preferred specification following guidance from the US Office of Management and Budget (*42*). Because many benefits accrue over long horizons while costs are incurred up front, estimated benefit-cost ratios are sensitive to assumptions regarding the persistence of realized reductions in flood-risk exposure (fig. S18).

The sensitivity analyses show that estimated benefit-cost ratios are sensitive to assumptions about the time horizon over which avoided damages persist and the discount rate used to value future benefits (fig. S19). This reflects the structure of buyout programs: Acquisition, demolition, and implementation costs are incurred up front, while benefits accrue gradually through avoided future building and content losses. As a result, shorter horizons and higher discount rates substantially reduce estimated benefit-cost ratios (fig. S18), particularly in the estimated move-effect and SFHA-exit scenarios. The one-at-a-time sensitivity analysis further highlights the importance of acquisition costs and damaging-flood recurrence for estimated net benefits (fig. S19). Benefit-cost ratios are reported across the full range of recurrence benchmarks to show how the estimated ratio varies with the assumed frequency of damaging floods (fig. S20).

The analysis should be interpreted as a stylized and conservative estimate of the benefits of buyout programs rather than a comprehensive social welfare analysis. The benefit calculation monetizes avoided building and content losses using observed NFIP payments as a conservative proxy for property damages and does not separately monetize uninsured losses, deductibles, displacement and temporary housing costs, emergency response costs, broader displacement or welfare effects, spillovers to neighboring properties, long-run ecosystem-service benefits, or potential changes in local development patterns. In addition, because some households would likely have reduced flood exposure even absent the program, the estimates should be interpreted as reflecting the incremental reductions in flood-risk exposure attributable to buyout participation rather than the full value of relocation itself. As a result, the preferred specification should be interpreted as a conservative estimate of the benefits of buyout programs under realized patterns of postbuyout mobility rather than an estimate of the maximum possible benefits under complete and permanent risk elimination.

### AI disclosure

AI (artificial intelligence) technologies (ChatGPT, OpenAI; Claude, Anthropic) were used to assist with code debugging and refinement and editing for clarity and presentation. All outputs were reviewed and verified by the author, who takes full responsibility for the content of the manuscript.

## Supplementary Materials

### This PDF file includes:

Supplementary Data Validation Figs. S1 to S26 Tables S1 to S6 References
