# Supplementary material for: Does government-assisted relocation help households
Source: Sci Adv. 2026 Nov 11;12(46):eaef4602. doi: 10.1126/sciadv.aef4602 (PMC13754018; doi:10.1126/sciadv.aef4602)
Supplement: Supplementary file 1 [file sciadv.aef4602_sm.pdf]

Science Advances
AAAS

Supplementary Materials for

Does government-assisted relocation help households

Rachel Young

Corresponding author: Rachel Young, rmyoung@umn.edu

Sci. Adv. **12**, eaef4602 (2026)
DOI: 10.1126/sciadv.aef4602

This PDF file includes:

Supplementary Data Validation
Figs. S1 to S26
Tables S1 to S6
References

Supplementary Data Validation

Validation and Measurement Checks

Representativeness of Infutor Several data checks were conducted to ensure data accuracy. Similar to prior studies, I find that the data is representative of the overall US adult population. (*47–49*) Fig. S23a–b shows the correlation between a 0.1% sample of the Infutor population and Census population in 1990, 2000, and 2010 at the state and county level. Over time, the completeness of Infutor data improves, showing consistently high correlations with census counts across the decades. The coefficients in Figs. S23a–b approach 0.001, equivalent to a perfect correlation between the census population and the 0.1% Infutor sample. These relationships also have very high R^2 (> 0.98) at both the state and county levels.

There are limitations: Infutor does not capture international moves; some demographic fields (e.g., birth dates) are missing for a subset of individuals; and homeowners and individuals with credit histories are overrepresented. Despite these limitations, the data remain one of the few nearly comprehensive address histories of U.S. adults.

Infutor under-counting of moves Infutor move counts correlate with administrative migration data but undercount moves relative to IRS Statistics of Income (SOI) county-to-county migration data. (*50, 51*) Infutor shows lower log odds of county out-migration compared to IRS data for 2000 and 2010, with differences ranging from 0.9 to 2 log odds (53% to 247% lower) after adjusting for population. One explanation is that IRS SOI includes the universe of tax filers, whereas Infutor relies more on credit-related data sources.

Infutor also undercounts moves relative to the American Community Survey (ACS). (*52*) The ACS reports when individuals moved to their current home (within one year, five years, ten years, etc.). Comparing these aggregates to the Infutor-derived likelihood of living in the buyout address prior to the buyout, Infutor undercounts moves within five years by 149% compared to the 2010 and 2015 ACS. This gap declines for moves within ten and fifteen years (79% and 43% undercounts, respectively), suggesting Infutor more reliably captures moves with longer lags.

To further assess whether Infutor mobility measures exhibit differential measurement error across neighborhood demographic groups, I additionally compare tract-level ACS and Infutor

mobility rates separately across census tract racial composition and income groups (Fig. S24). Because the ACS mobility measure is based on self-reported residential mobility within the past year, while the Infutor measure is derived from observed address changes, the two measures are not directly comparable in levels. Nevertheless, ACS mobility rates are generally higher than Infutor-derived mobility rates, and the relationship between the two measures appears broadly similar across subgroup definitions.

These discrepancies are consistent across treated and control groups (Table S4).

FEMA data quality + non-compliance I verified the NPR buyout count against FEMA's publicly available data, OpenFEMA (*10*). OpenFEMA is an online platform that hosts downloadable datasets including descriptions of disaster declarations, program spending at the project and property levels, community grant applications, and NFIP information. FEMA released these data to "promote transparency, reduces complexity related to public data requests, and improves how stakeholders interact with and understand the program". (*10*) OpenFEMA includes anonymized property- and project-level information on participants in all of FEMA's hazard mitigation grant programs. I compared the count and timing of properties reported as "property acquisitions" in the OpenFEMA against the NPR FOIA data, and the decade-level counts of the number of buyouts is similar (Fig. S2).

Public FEMA data records have several observable issues and errors evident in the OpenFEMA dataset (e.g., incorrect zip codes and misspelled state names), stemming from underlying problems with FEMA's record-keeping. A DHS OIG audit of the program data found FEMA hazard mitigation grant program records to be incomplete, inaccurate, and sometimes noncompliant with open space requirements. AECOM's digitization effort documented missing parcel data, incorrect addresses, and thousands of properties still containing structures decades after acquisition.

My own checks yield consistent evidence of underlying record issues: (1) 15% of buyout locations still show structures in Google satellite imagery (Fig. S25); (2) 14% have addresses that cannot be located or are not mapped. These issues contribute to apparent low move rates among treated individuals and are incorporated into the adjusted estimates used in the benefit–cost analysis.

Adjusted move-rate calculations Because Infutor undercounts moves, I report here adjusted estimates that scale the observed treated-group cumulative move rates using the ratio between ACS- and Infutor-measured mobility rates in the corresponding comparison populations. Specifically, I calculate the adjusted treated-group move rate as

$$\widetilde{M}_t^T = M_t^{T,\text{Infutor}} \times \frac{M^{\text{ACS}}}{M^{\text{Infutor}}},$$

where $M_t^{T,\text{Infutor}}$ is the observed cumulative move rate among treated households in Infutor, and $M^{\text{ACS}}/M^{\text{Infutor}}$ is the proportional discrepancy between ACS- and Infutor-measured aggregate move rates (Fig. S25; Fig. S26; Table S4). The additional adjustment for FEMA non-compliance and data inaccuracies is then incorporated additively using the estimated share of properties with incorrect buyout status or unresolved structures based on DHS OIG findings, AECOM validation efforts, and my own manual verification sample.

Using the ACS adjustment alone, the estimated likelihood of moving to a new address after the buyout approval (treated group) becomes:

- 56.3% within five years
- 71.8% within fifteen years

Including FEMA non-compliance and data errors further increases the adjusted move-rate. The DHS OIG audit revealed widespread data inaccuracies in FEMA records, and AECOM's digitization identified 8,000 properties with incorrect or missing parcel data and 2,800 properties that still had structures present despite being labeled as buyouts. (*15*) My own random sampling found that approximately 15% of listed buyout locations still had structures (Fig. S25). An additional 14% of "removed" properties had non-searchable or nonexistent addresses.

Accounting for both Infutor undercounting and FEMA non-compliance:

- 74.3% likelihood of moving after five years
- 74% likelihood after ten years (56% Infutor-adjusted move rate + 18% estimated FEMA non-compliance contribution)

These calculations are intended as approximate benchmarking exercises rather than formal causal estimates, but they suggest that the observed Infutor-based move rates likely understate the true share of households that ultimately relocate following buyout approval.

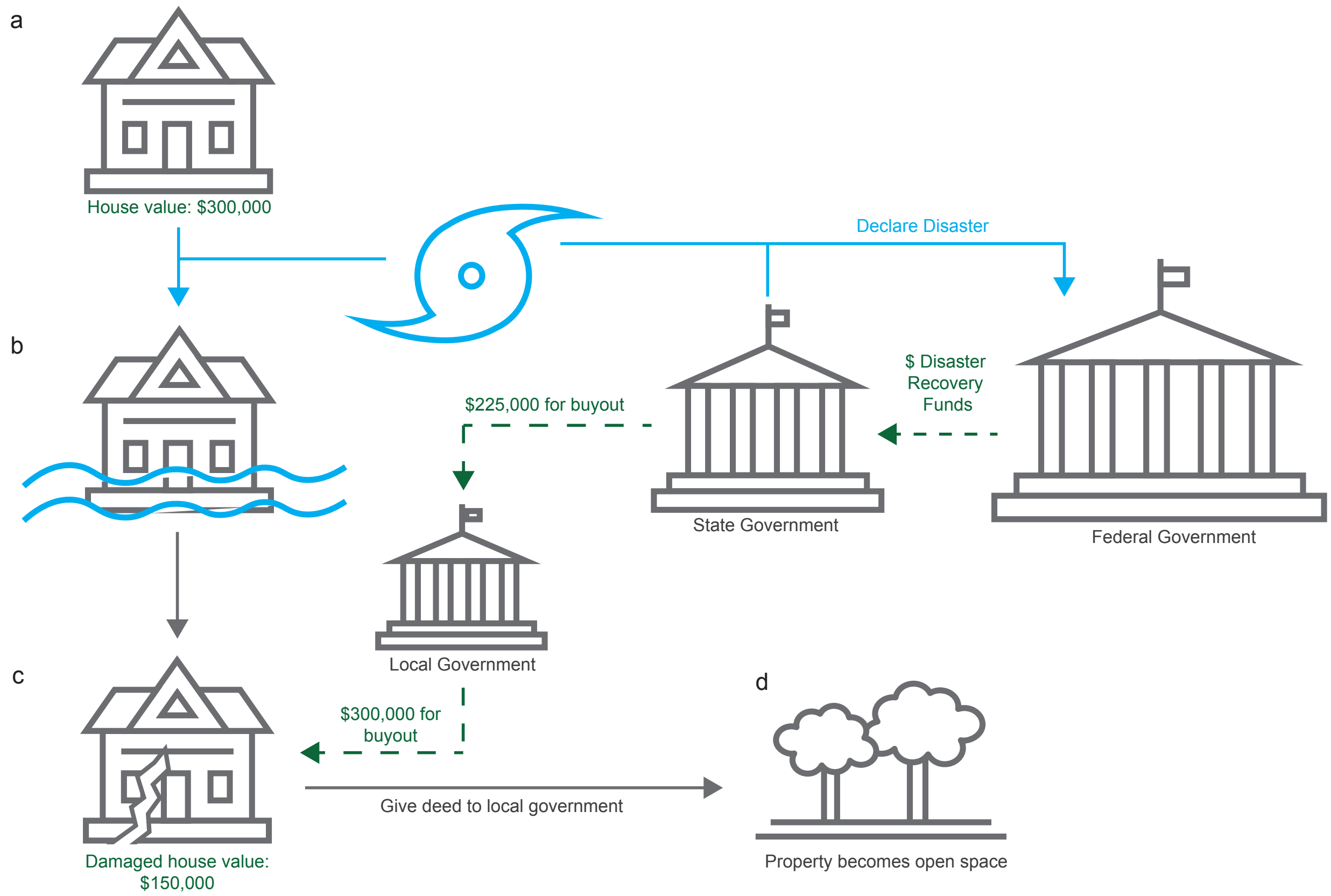

Figure S1: **Diagram of the FEMA buyout process through the Hazard Mitigation Grant Program.** (**a**) Pre-disaster home in the Special Flood Hazard Area (SFHA) with a market value of $300,000. (**b**) A hurricane event causes the home to flood, triggering the Governor's disaster declaration request. (**c**) Following a Presidential Disaster Declaration, the federal government allocates disaster-relief funding. The state solicits applications from local governments and allocates 75% of the pre-disaster market value for the buyout ("$225,000"). Local governments provide the remaining cost share ("$300,000 for buyout"). The damaged structure is valued at 50% of its pre-disaster value ("$150,000"). (**d**) The property owner transfers the deed to the local government; the house is demolished and converted to open space. *Illustration credit: Created by the author using Adobe Illustrator.*

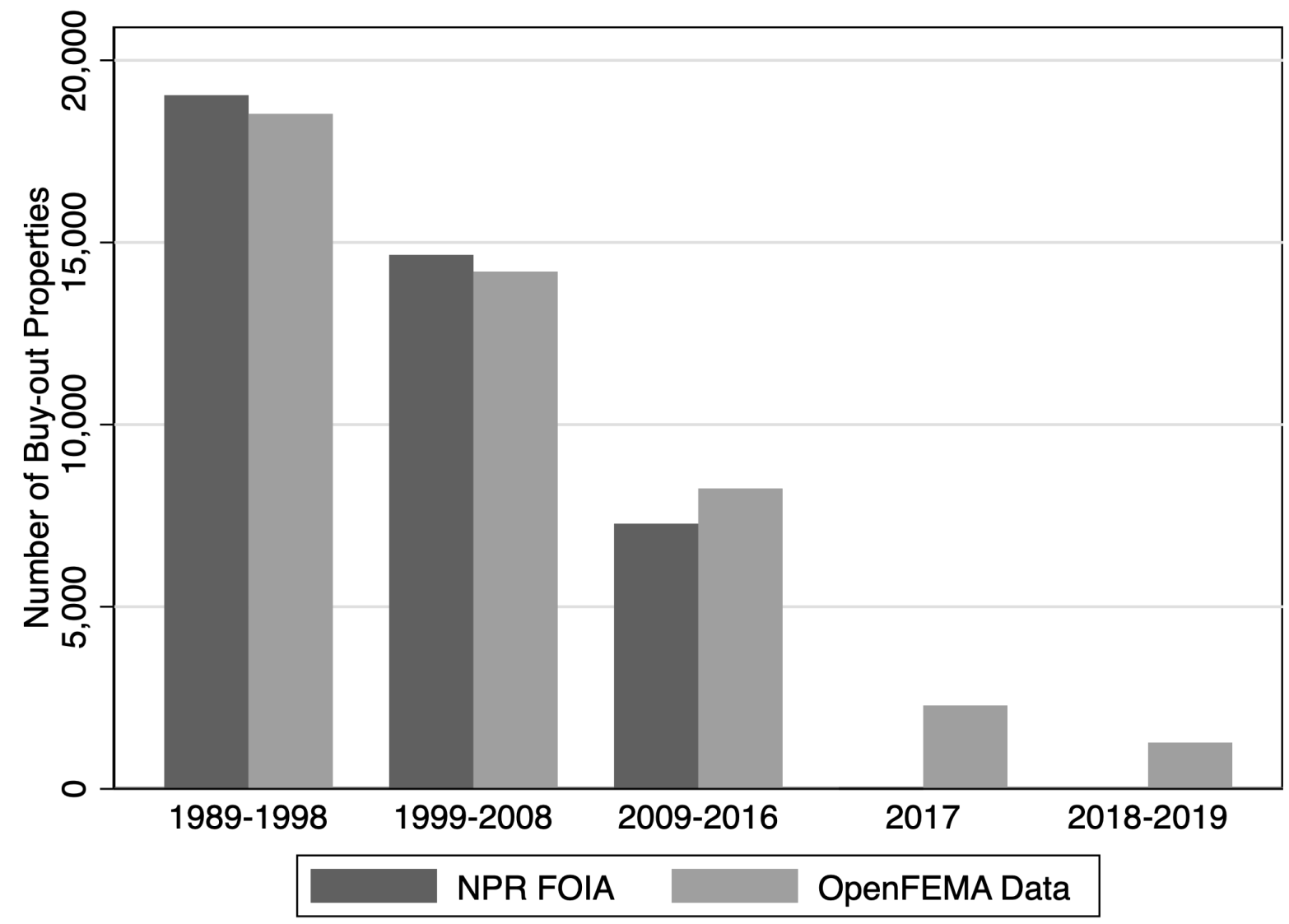

Figure S2: **Comparing NPR and OpenFEMA buyout property data.** Counts of buyout properties per decade in the NPR FOIA dataset (dark gray) and the OpenFEMA dataset (light gray) (*10*).

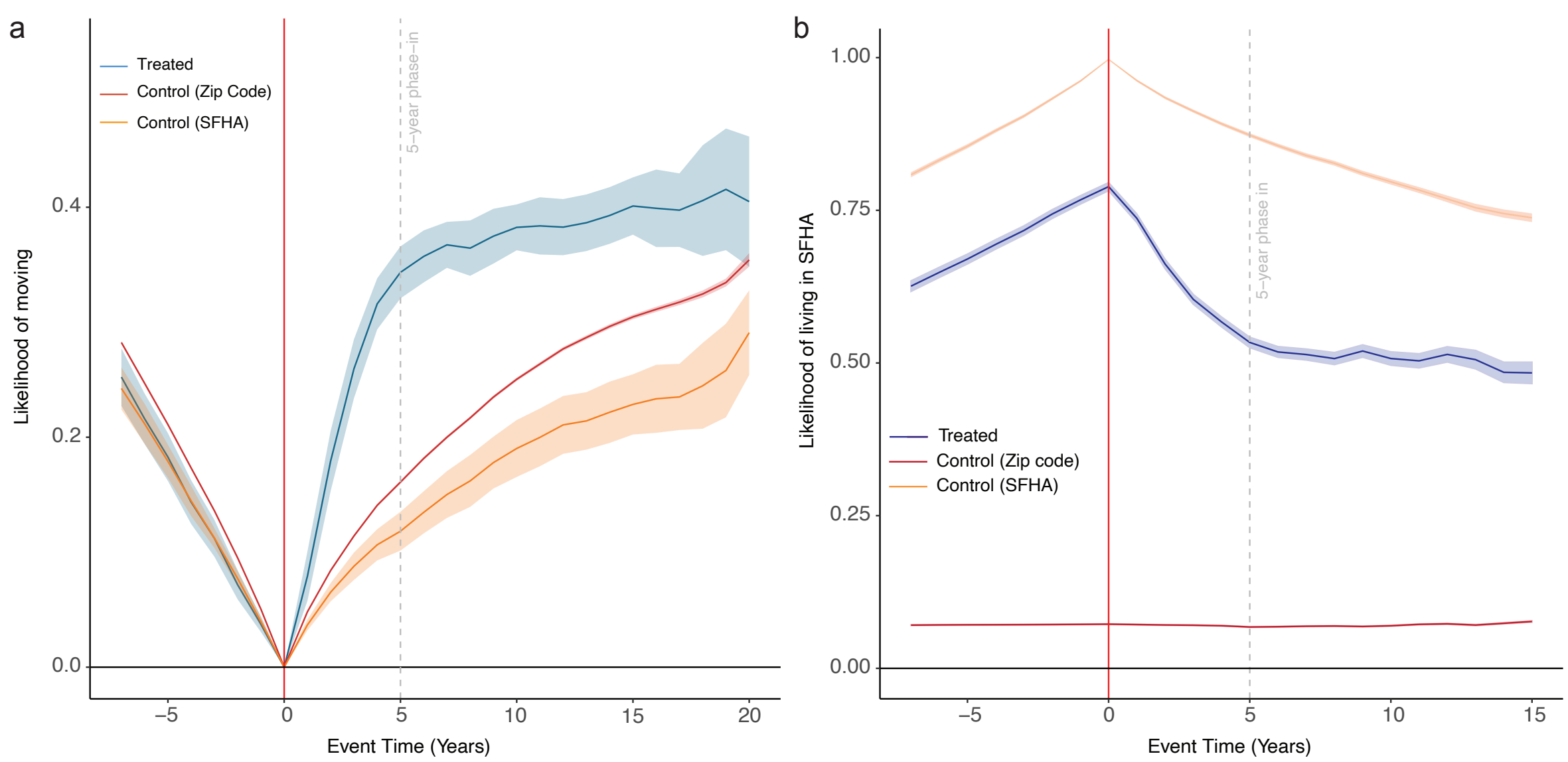

Figure S3: **Cumulative average likelihoods of moving pre- and post-buyout.** (**a**) Cumulative effect of the buyout on the likelihood of living at a non-buyout address for treated individuals (blue) and two control groups: (1) a random subset of individuals living in the buyout ZIP code in the buyout year (red), and (2) those individuals who also reside in SFHAs (yellow). (**b**) Cumulative effect of the buyout on the likelihood of living in an SFHA. Shaded areas show 95% confidence intervals.

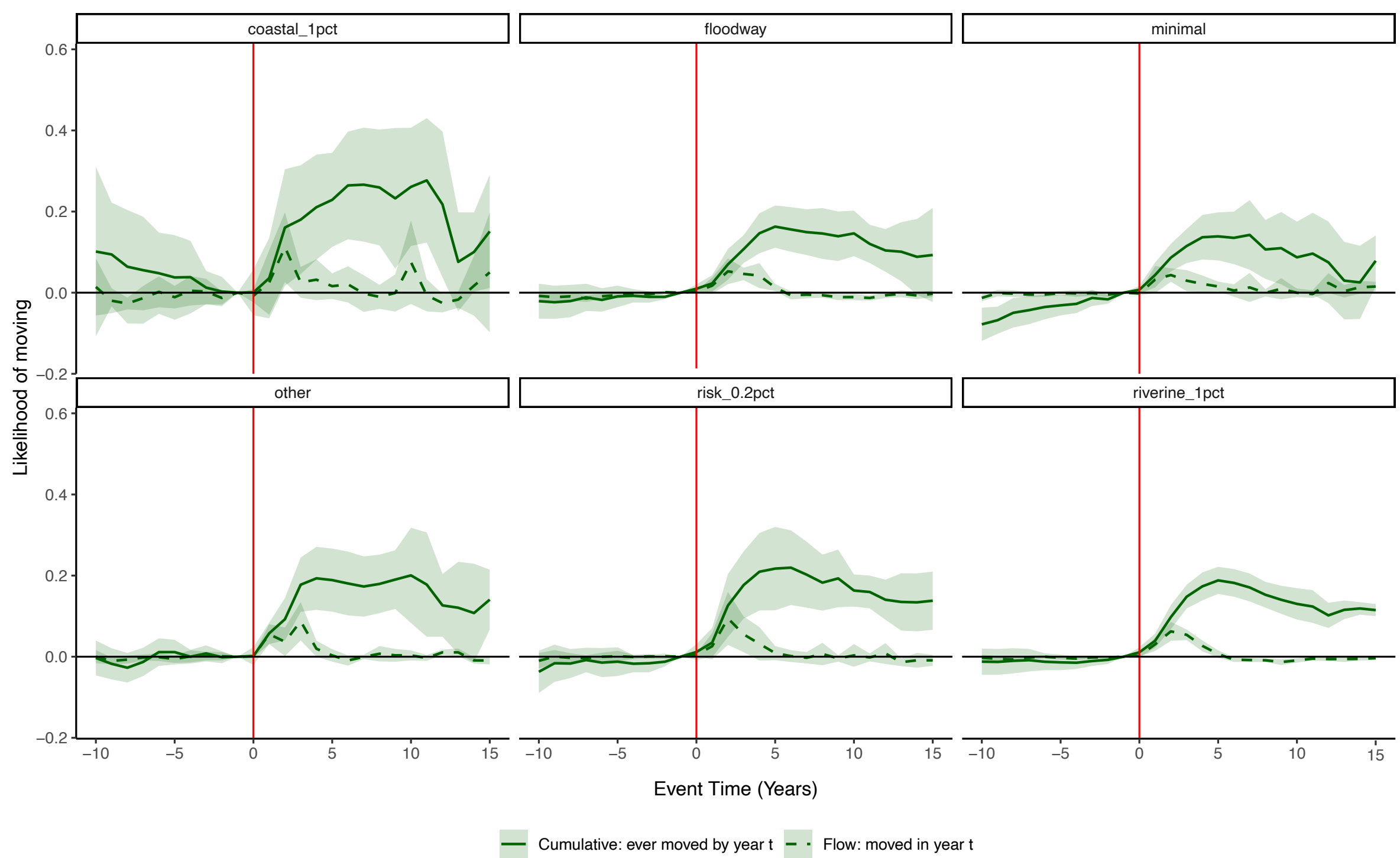

Figure S4: **Cumulative vs. annual move effects by baseline flood-risk group.** Estimated cumulative and annual effects of participation in the buyout program on residential mobility, by baseline flood-risk category. Flood-risk categories are constructed using FEMA flood zone classifications fixed at each property's event year ($\tau = 0$). The cumulative estimates measure the probability of ever moving by year t, while the annual flow estimates measure the probability of moving in a given year. Results are shown separately for coastal 1% floodplains, riverine 1% floodplains, 0.2% annual-chance floodplains, floodways, minimal-risk areas, and other flood-risk classifications. Shaded regions indicate 95% confidence intervals clustered at the state level.

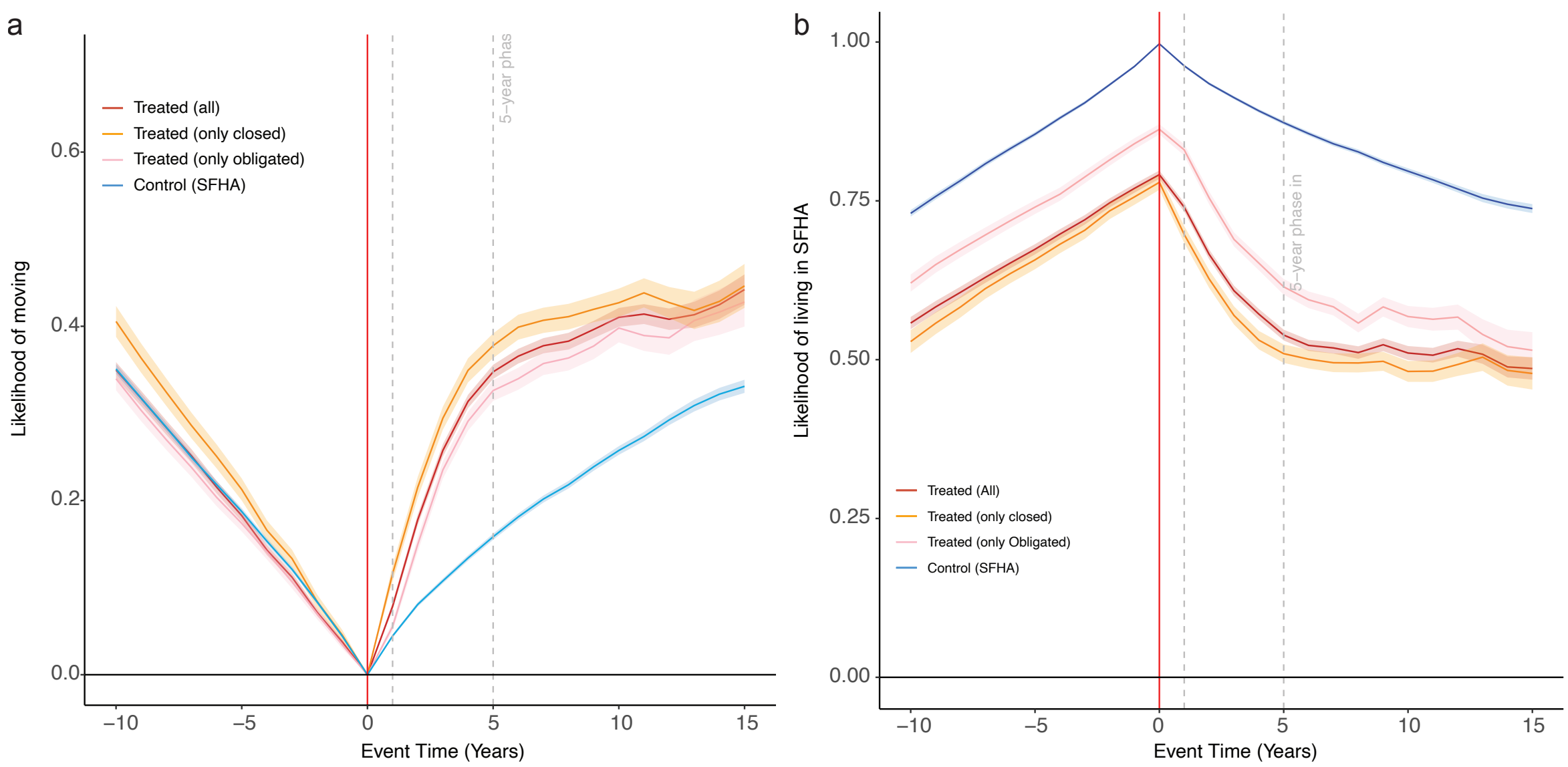

Figure S5: **Cumulative average likelihoods of moving by FEMA project status.** (**a**) Cumulative likelihood of moving for treated individuals, disaggregated by FEMA marking: full set of buyouts (red), "closed" buyouts (yellow), and "obligated" buyouts (pink), compared with SFHA controls (blue). (**b**) Cumulative likelihood of living in an SFHA for the same groups. Shaded areas show 95% confidence intervals.

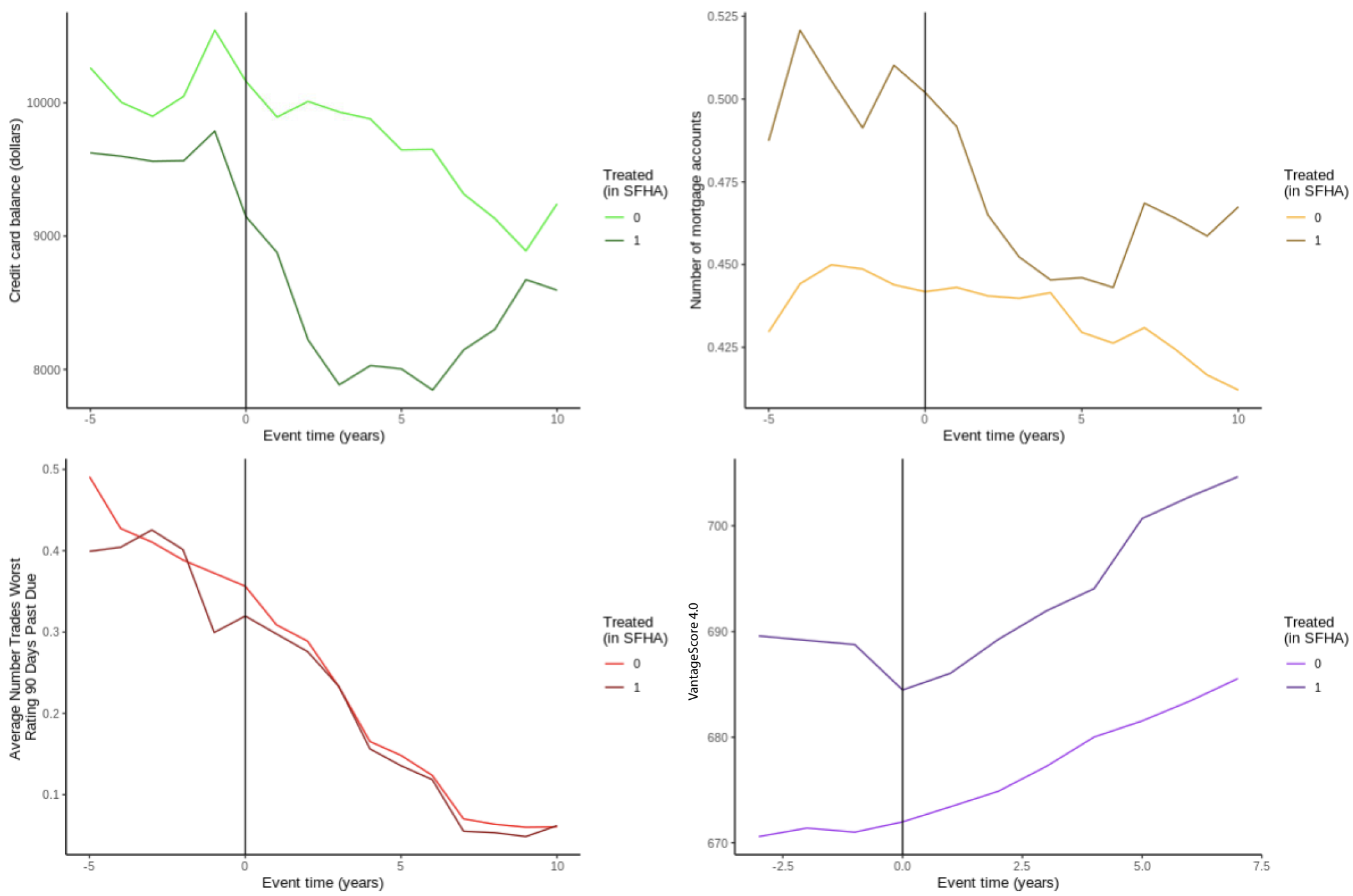

Figure S6: **Average financial outcomes before and after the buyout, treated and control groups.** Mean financial outcomes for treated individuals and SFHA control individuals in event time. (**a**) Credit card debt. (**b**) Number of mortgages. (**c**) Number of trades with a worst rating of 90 days past due. (**d**) VantageScore 4.0 credit score. Shaded areas show 95% confidence intervals.

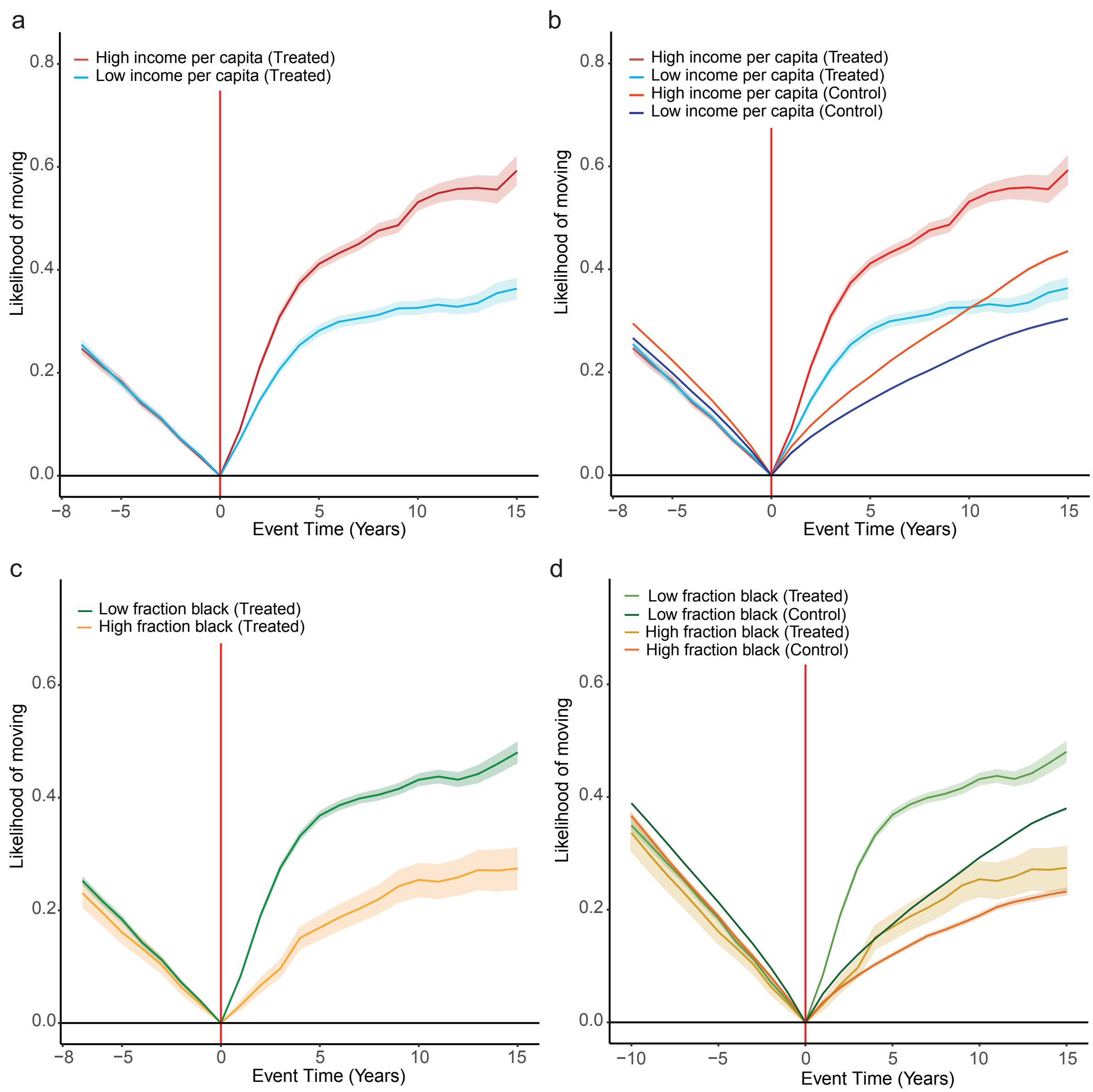

Figure S7: **Differential effect of the buyout on moving for the treated and the control groups by neighborhood race and income composition**. Cumulative effects of the buyout on moving to a new address for the treated and control groups and 95% C.I. (**a**) Effects for buyout participants living in high (≥median) and low (<median) income per capita census tracts prior to the buyout. (**b**) Estimated moving effect for treated and control individuals living in high and low income census tracts prior to the buyout. (**c**) Effects for the buyout participants ("Treated") living in (pre-buyout) high-fraction (≥50%) and low-fraction (<50%) Black population census tracts. (**d**) Effects for the treated and the control individuals living in the high and low-fraction Black population census tracts.

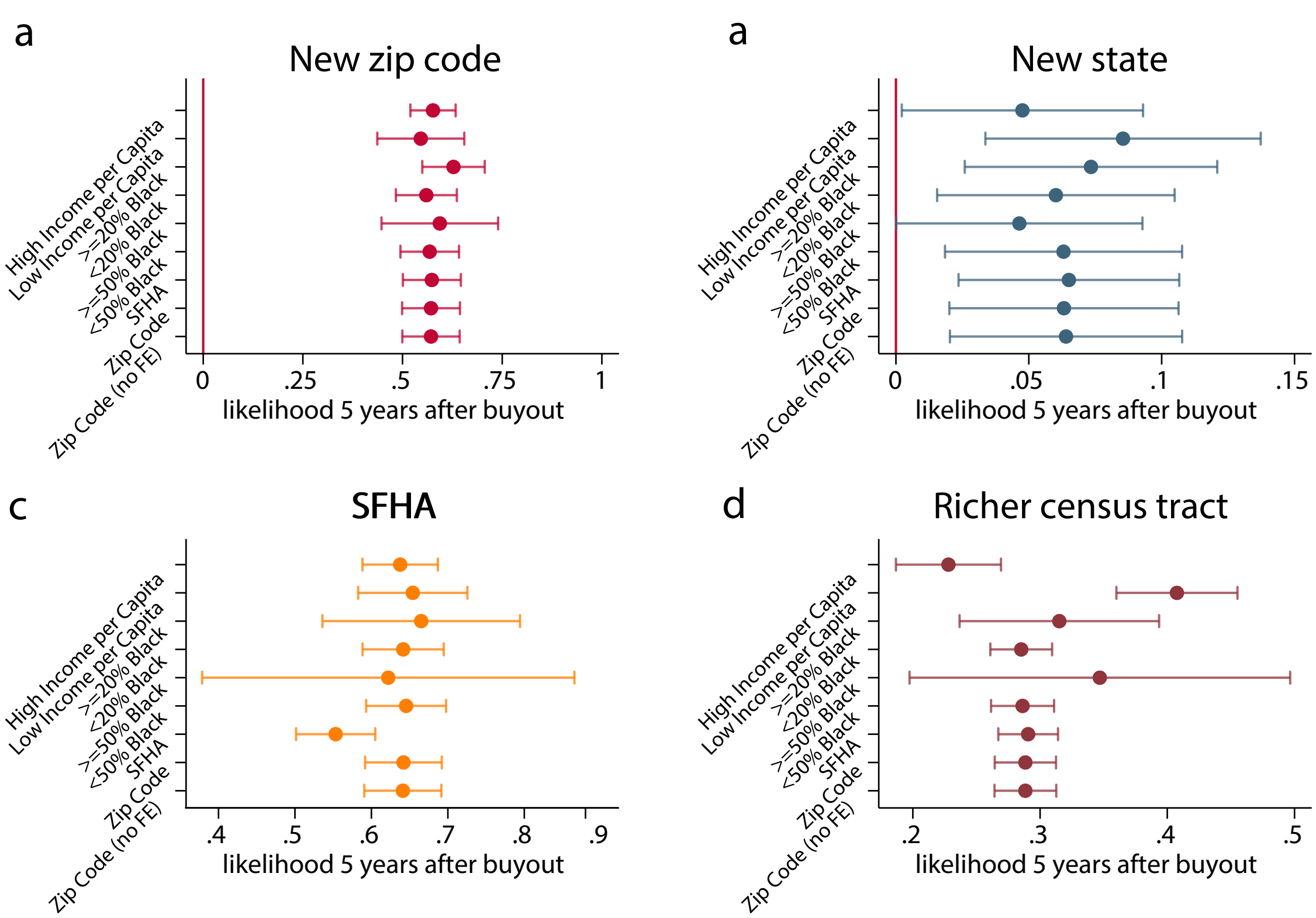

Figure S8: **Five-year buyout effects among households that relocate.** Estimated cumulative effects five years post-buyout relative to three control definitions. Heterogeneous effects shown for high/low income tracts and high/low fraction Black tracts. Panels: (**a**) new ZIP code; (**b**) new state; (**c**) out of SFHA; (**d**) higher-income tract. Whiskers show 95% confidence intervals.

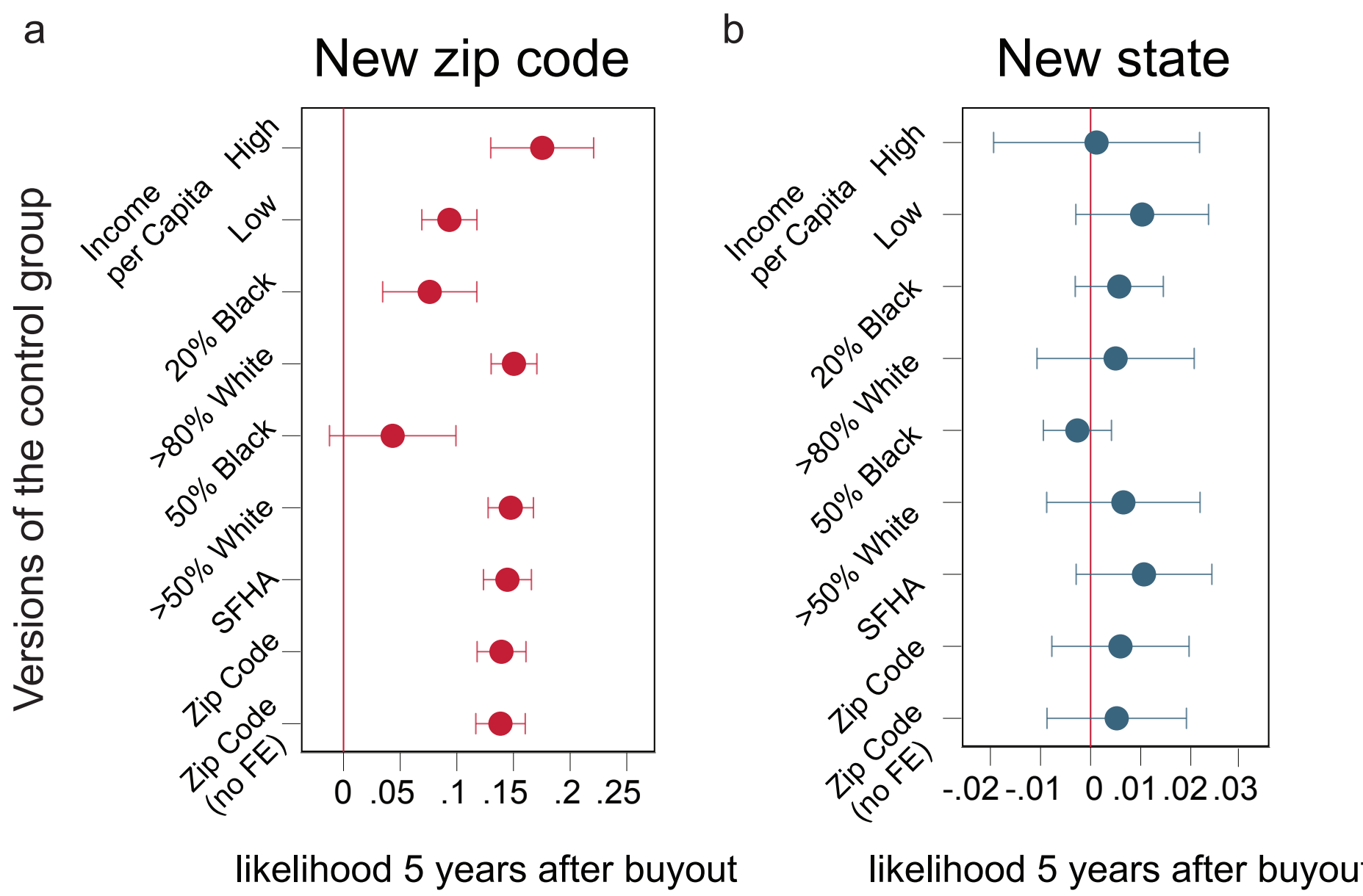

Figure S9: **Five-year buyout effects on moving to a new ZIP code or a new state.** Estimated cumulative effects five years post-buyout relative to three control definitions: (1) individuals in the buyout ZIP code ("ZIP Code (no FE)"), (2) individuals in the buyout ZIP code and same state–year cohort ("ZIP Code"), and (3) individuals in the buyout ZIP code, same state–year cohort, and in SFHAs ("SFHA"). Heterogeneous effects are shown for census tracts with high vs. low Black population shares and high vs. low income. Whiskers show 95% confidence intervals.

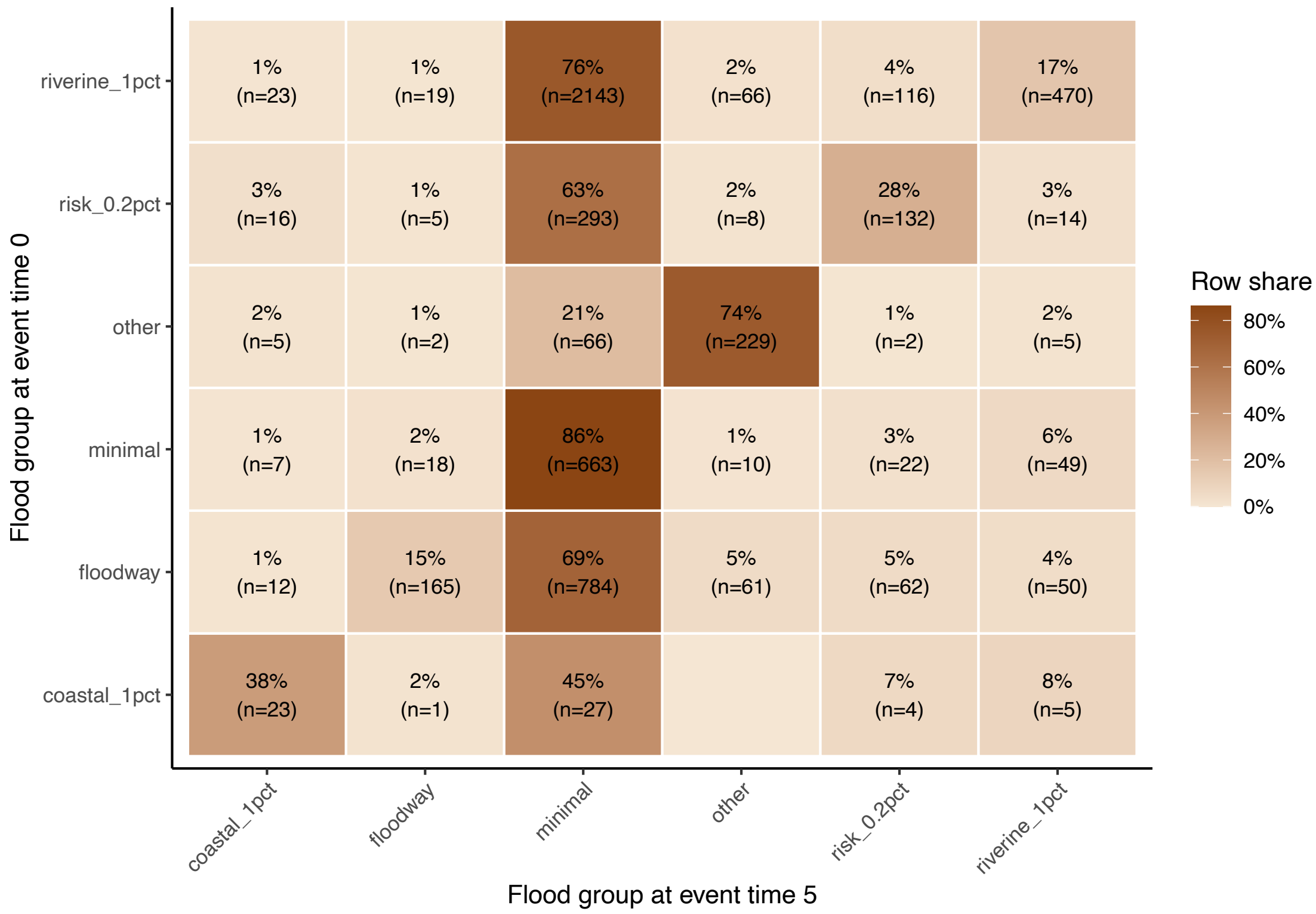

Figure S10: **Flood-group transitions among treated mover properties.** Flood-group transitions among treated households that relocate between event time 0 and event time 5. Rows indicate the FEMA flood-risk category of the origin property at event time 0, while columns indicate the flood-risk category of the destination property at event time 5. Cell values report row percentages, with rows summing to 100% within each origin flood group.

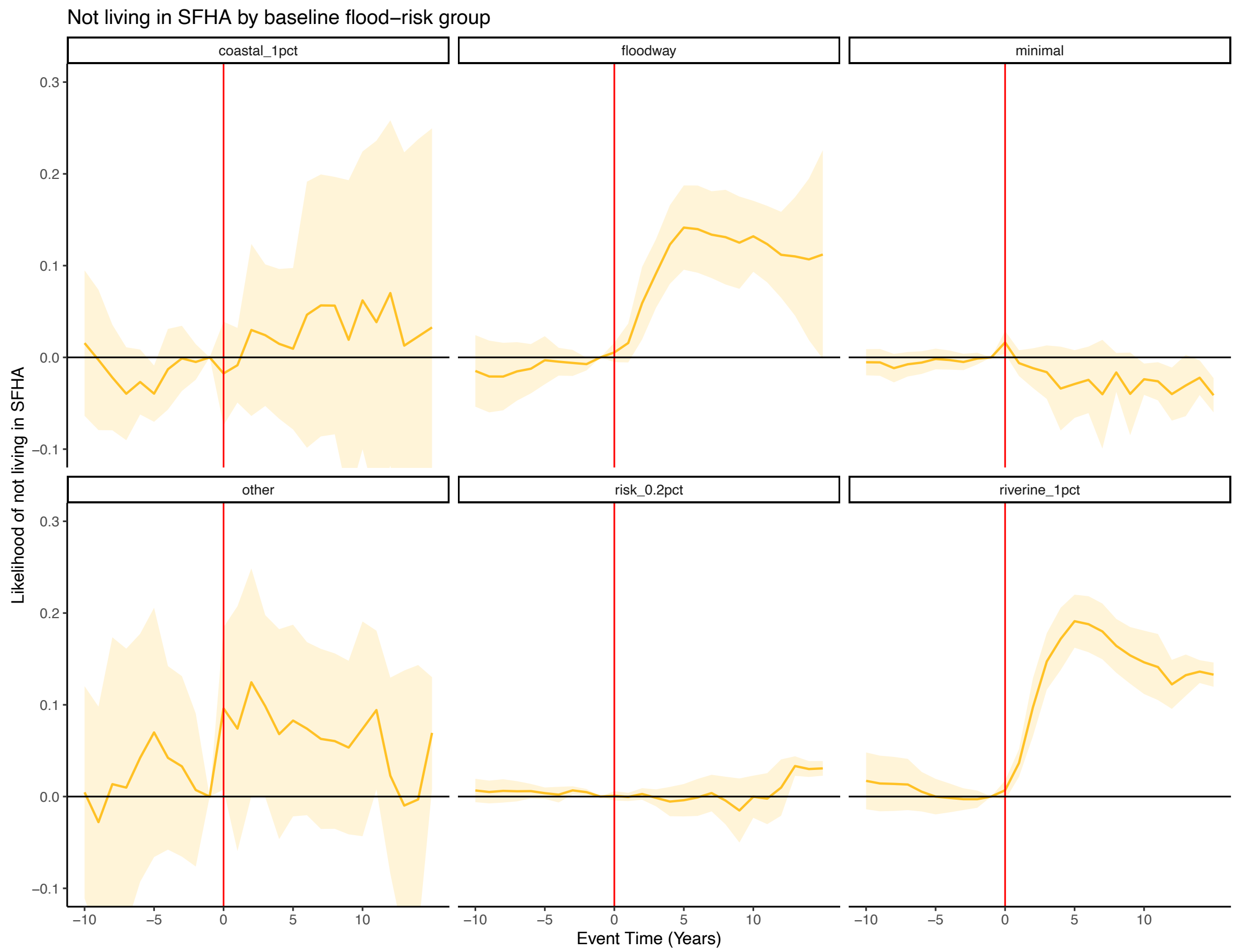

Figure S11: **Not living in SFHA by baseline flood-risk group.** Estimated effects of participation in the buyout program on the probability of no longer residing within a FEMA Special Flood Hazard Area (SFHA), by baseline flood-risk category. Flood-risk categories are constructed using FEMA flood zone classifications fixed at each property's event year ($\tau = 0$). Estimates are shown separately for coastal 1% floodplains, riverine 1% floodplains, 0.2% annual-chance floodplains, floodways, minimal-risk areas, and other flood-risk classifications. Shaded regions indicate 95% confidence intervals clustered at the state level.

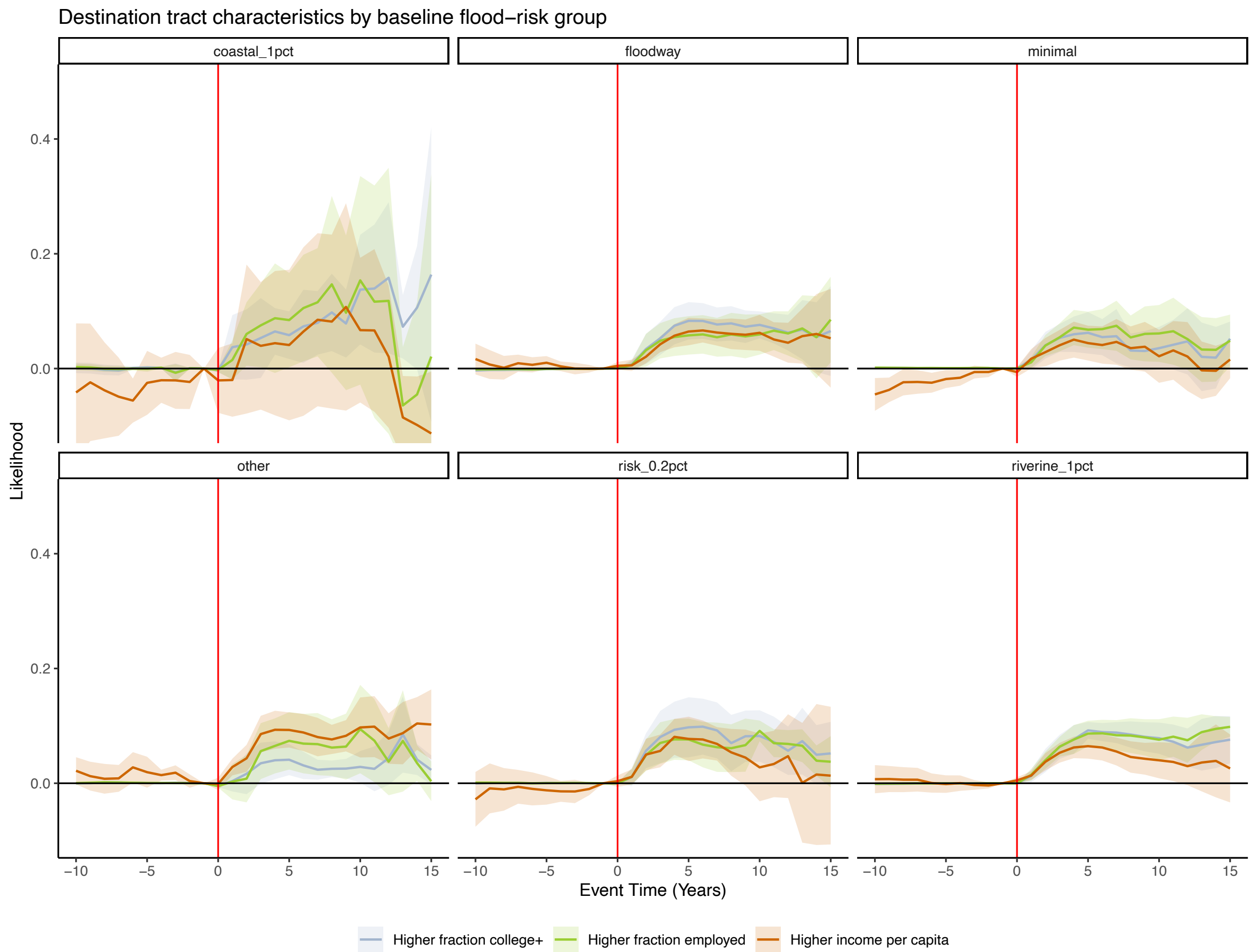

Figure S12: **Destination neighborhood characteristics by flood-risk group.** Estimated effects of participation in the buyout program on destination neighborhood characteristics, by flood-risk category. Outcomes include the likelihood of relocating to a census tract with higher per-capita income, higher employment rates, and higher shares of college-educated residents relative to the origin tract. Flood-risk categories are constructed from FEMA flood zone classifications and include coastal 1% floodplains, riverine 1% floodplains, 0.2% annual-chance floodplains, floodways, minimal-risk areas, and other flood-risk classifications. Vertical red lines indicate the buyout event year ($\tau = 0$). Shaded regions indicate 95% confidence intervals clustered at the state level.

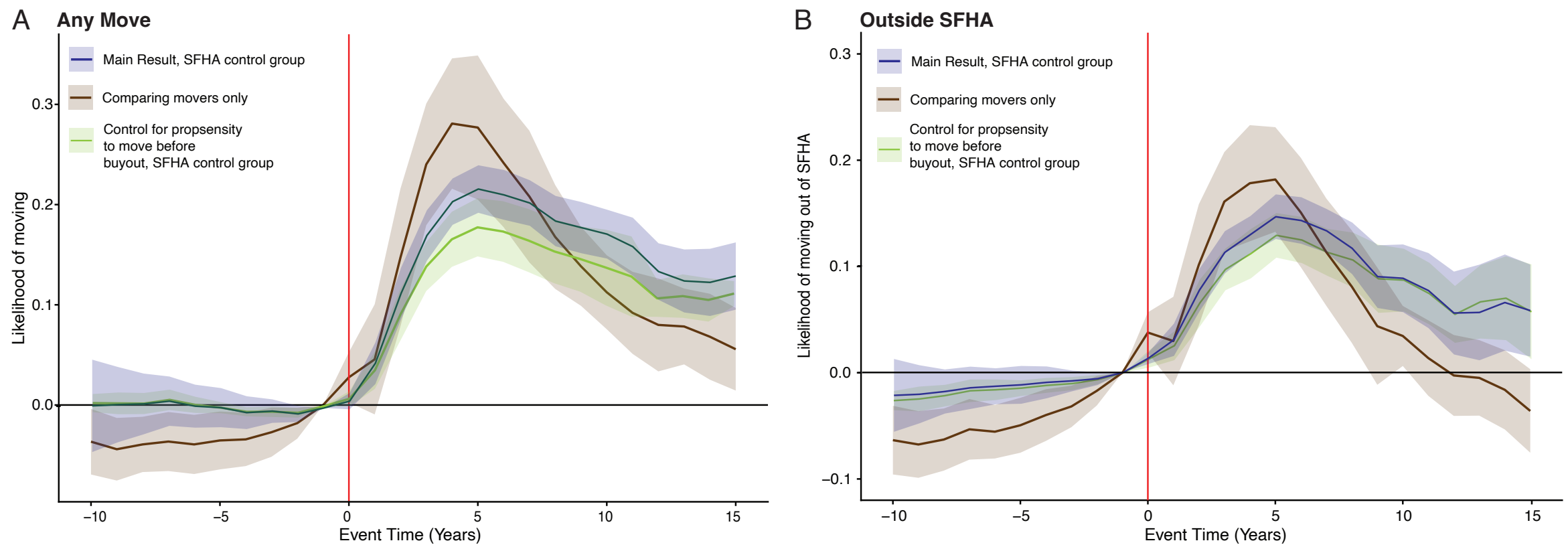

Figure S13: **Estimated cumulative effect of buyout participation on the probability of relocating under four alternative specifications.** (**a**) Estimated cumulative effects of participation in the buyout program on residential mobility. (**b**) Estimated cumulative effects on living outside of the SFHA area. Blue lines reproduce the main specification. Brown lines restrict the analysis to households observed relocating after treatment ("movers only"). Green lines additionally control for pre-treatment moving propensity. Shaded regions indicate 95% confidence intervals.

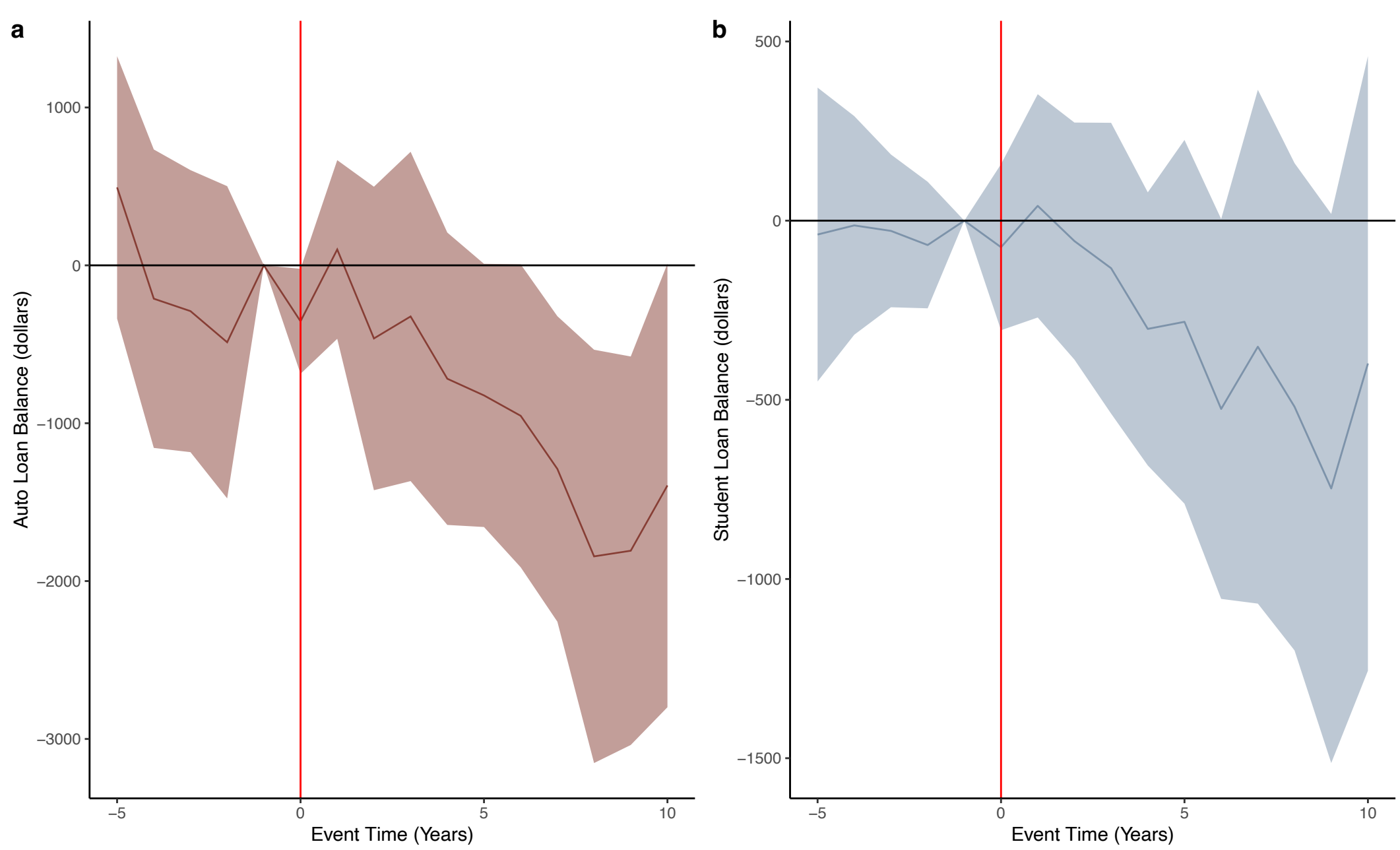

Figure S14: **Effect of buyouts on auto and student loan balances.** Estimated marginal effects up to ten years after the buyout. (**a**) Auto loan balances. (**b**) Student loan balances. Shaded areas are 95% confidence intervals.

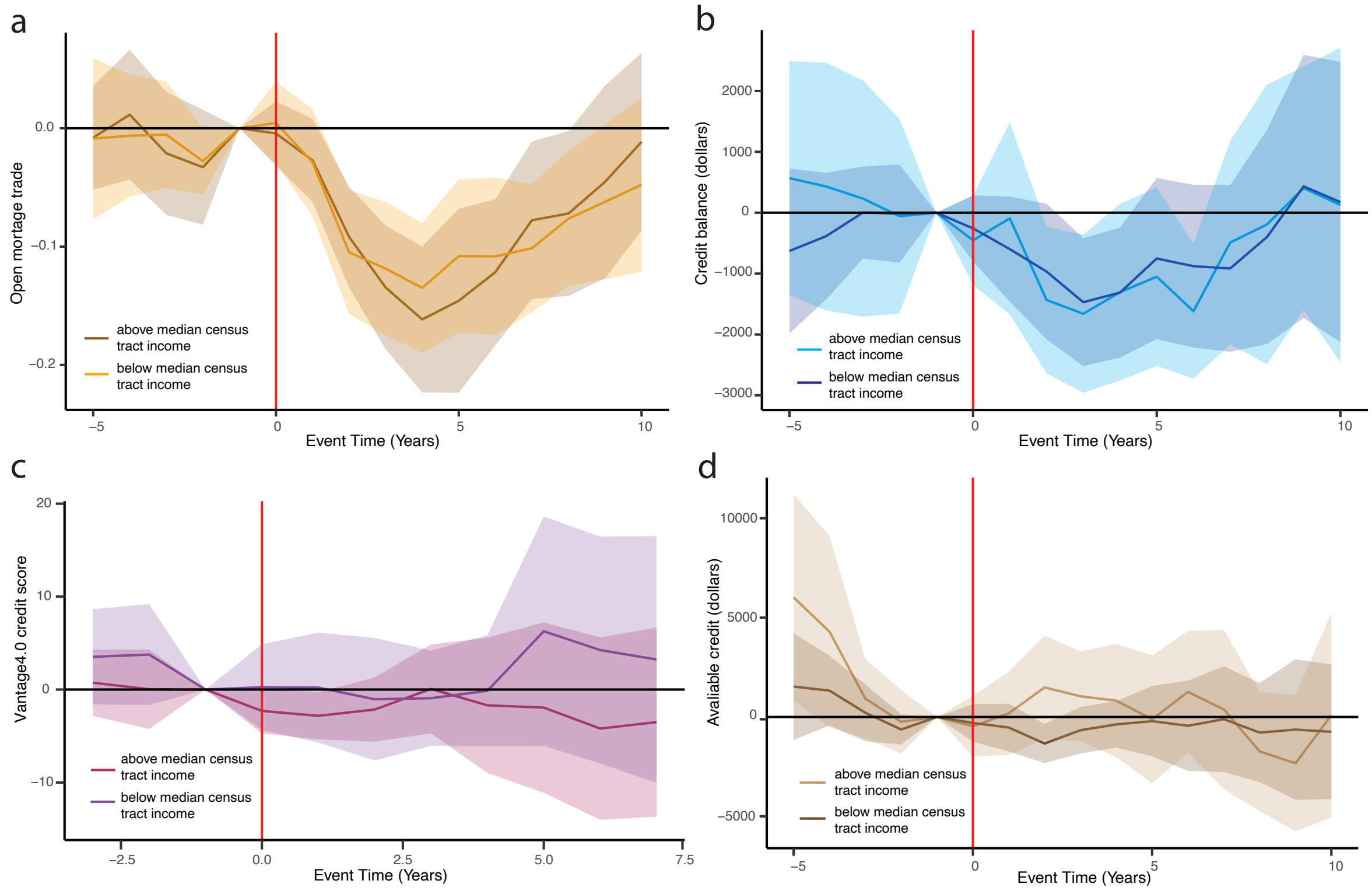

Figure S15: **Heterogeneous effects of buyouts by census tract characteristics.** Effects separately estimated for high vs. low income tracts and high vs. low fraction-Black tracts. (**a**) Mortgages. (**b**) Credit card balances. (**c**) VantageScore 4.0 credit scores. (**d**) Available credit. Shaded areas show 95% confidence intervals.

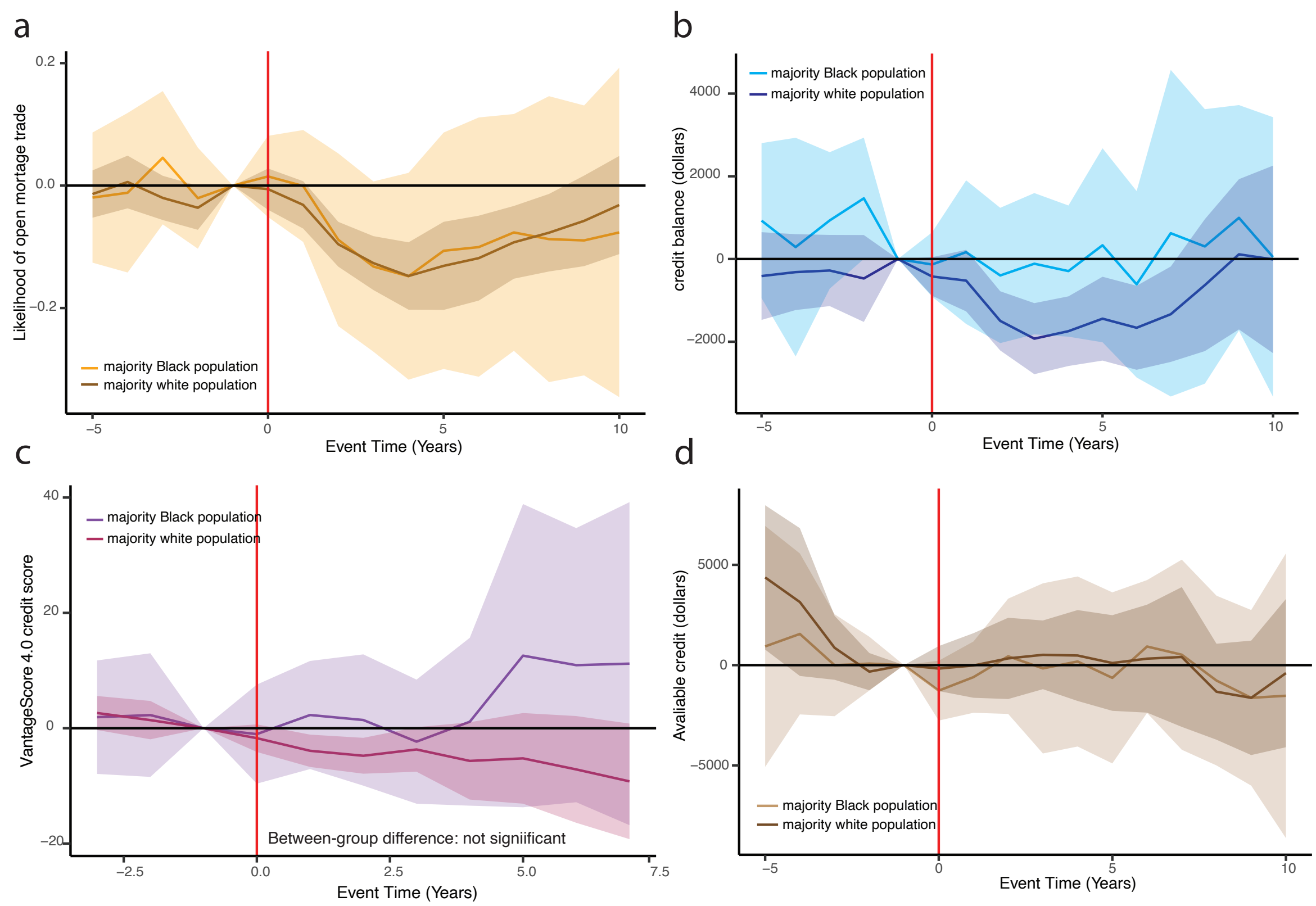

Figure S16: **Differential effect of the buyout by neighborhood racial composition.** Effects of the buyout on individuals living in census tracts with relatively higher or lower shares of Black residents, relative to comparable individuals. Color bands indicate 95% confidence intervals. (**a**) Impacts on number of mortgages. (**b**) Impacts on credit card balances (dollars). (**c**) Impacts on VantageScore 4.0 credit scores. Differences between the estimated effects for higher- and lower-share Black census tracts are not statistically significant. (**d**) Impacts on available credit (dollars).

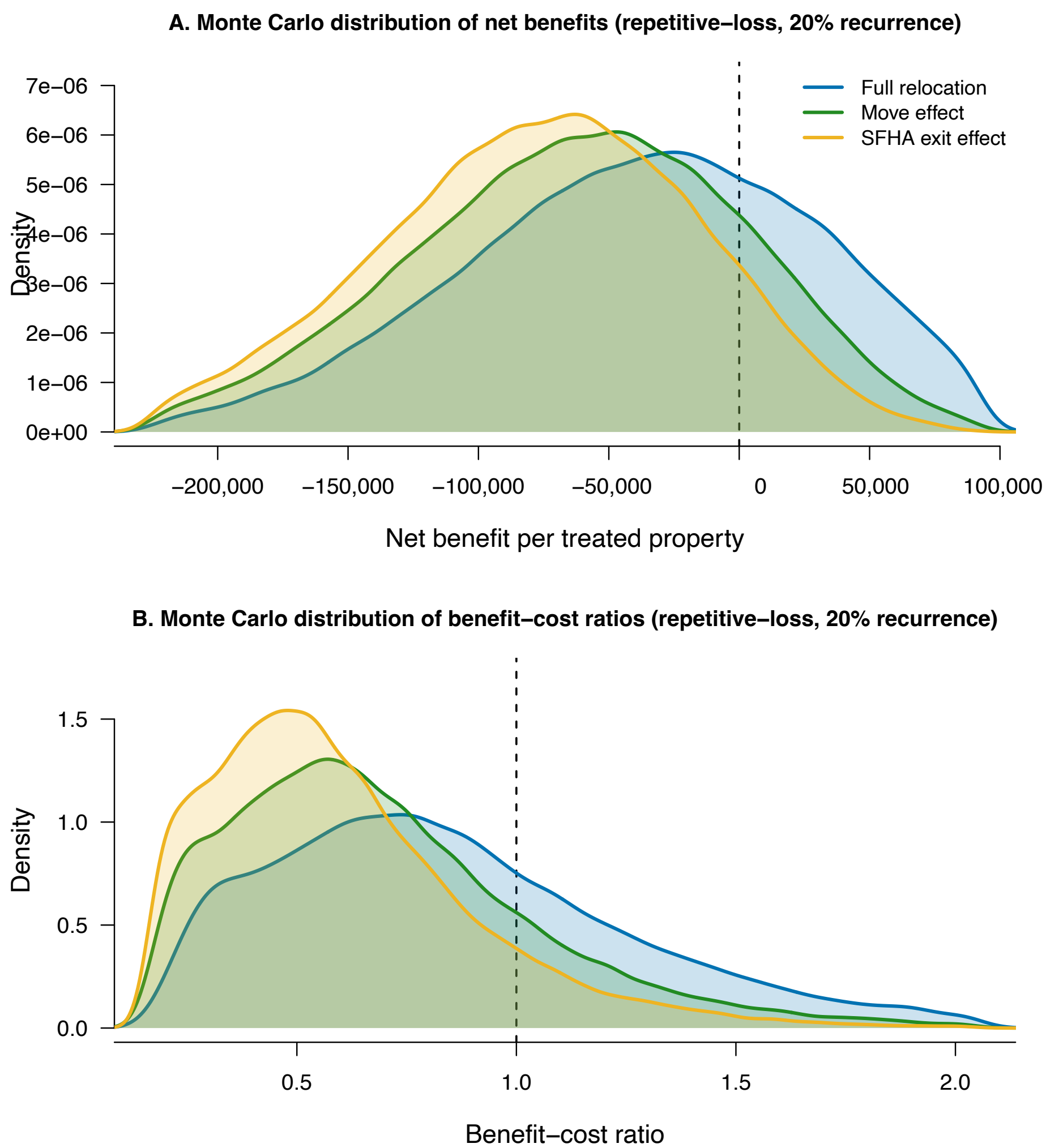

Figure S17: **Monte Carlo distributions of estimated net benefits and benefit–cost ratios.** **(a)** Distribution of estimated net benefits per treated property across Monte Carlo simulations under the three buyout scenarios, at the repetitive-loss (20%) recurrence benchmark. Simulations jointly vary acquisition costs, demolition and site-restoration costs, the risk-reduction effect, the conditional building-and-contents loss, disaster assistance expenditures, the open-space amenity co-benefit, discount rates, and the benefit horizon. Vertical dashed lines indicate zero net benefits. **(b)** Distribution of estimated benefit–cost ratios across Monte Carlo simulations under the three buyout scenarios. Vertical dashed lines indicate a benefit–cost ratio of one.

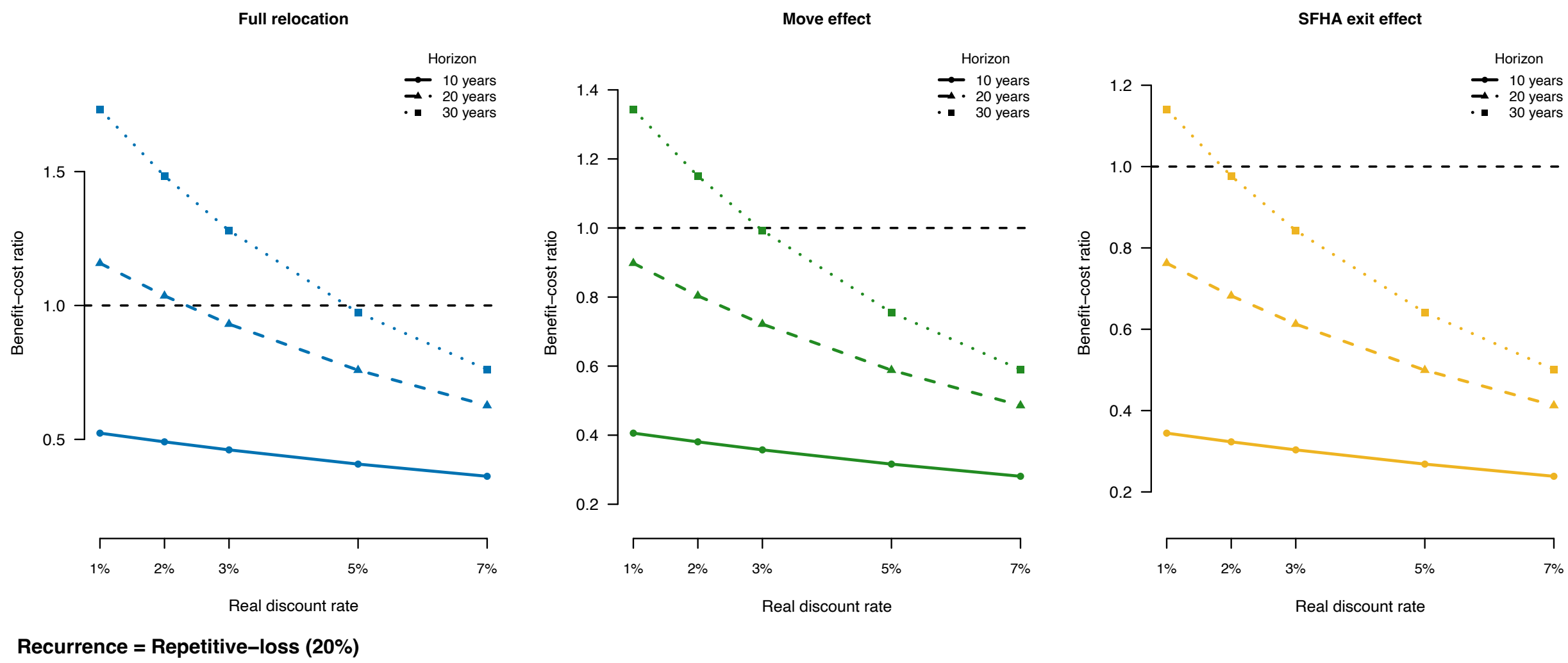

Figure S18: **Sensitivity of estimated benefit–cost ratios to discount rates and benefit time horizons.** Estimated benefit–cost ratios under alternative persistence of avoided flood-related losses and associated benefits following buyout participation, at the repetitive-loss (20%) recurrence benchmark. Results are shown for real discount rates ranging from 1% to 7% and for alternative benefit horizons of 10, 20, and 30 years under the three buyout scenarios. Horizontal dashed lines indicate a benefit–cost ratio of one.

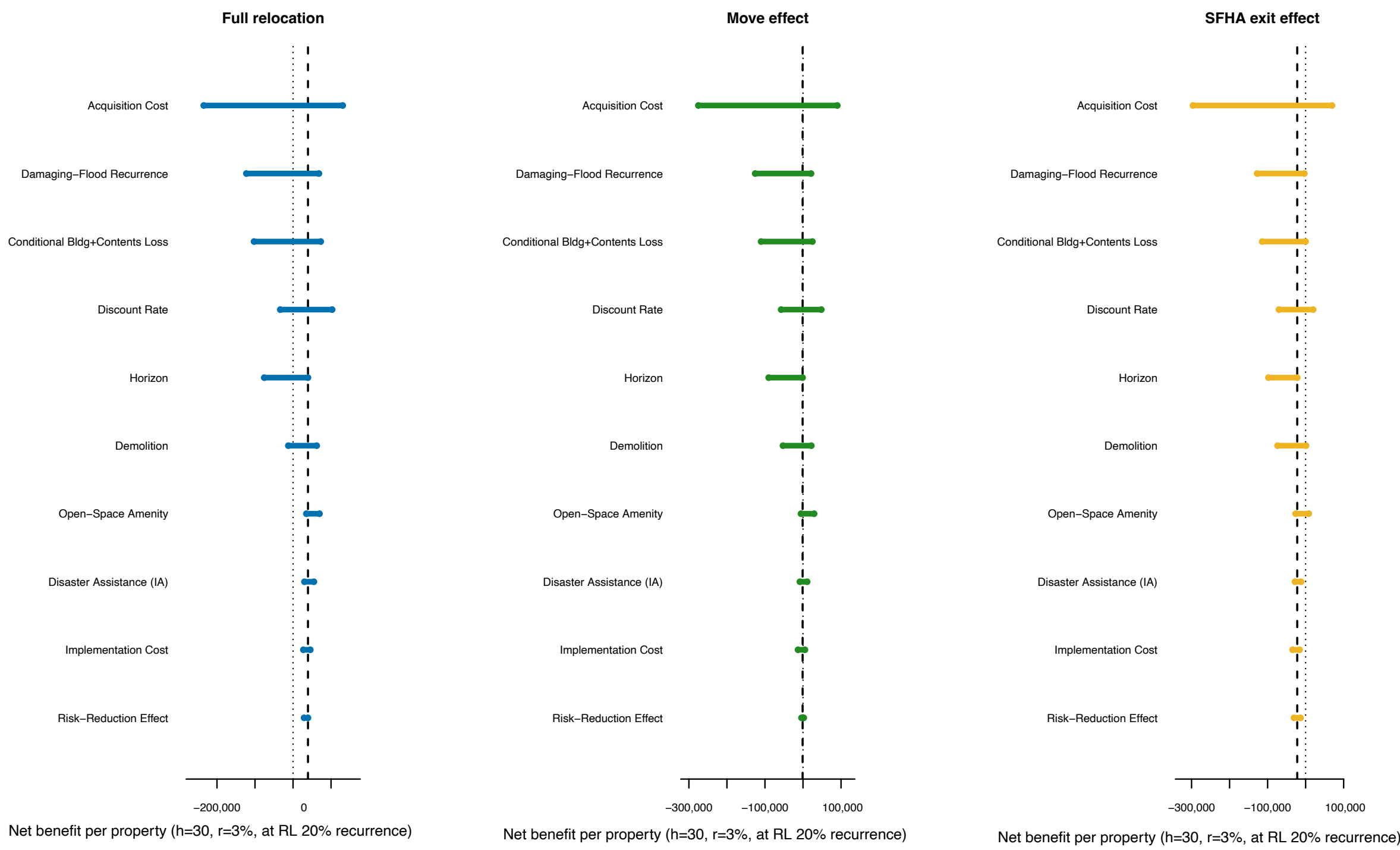

Figure S19: **One-at-a-time sensitivity analysis of estimated net benefits.** Rank plot of sensitivity of estimated per-property net benefits to variation in acquisition costs, demolition and site-restoration costs, damaging-flood recurrence, conditional building-and-contents loss, implementation costs, risk-reduction effect, disaster assistance expenditures, open-space amenity co-benefit, discount rates, and benefit horizon, under the three benefit–cost analysis scenarios (repetitive-loss recurrence benchmark of 20%). Horizontal lines represent the change in estimated net benefits when a single parameter is varied between its low and high values while all other assumptions are held fixed at their preferred values. Dashed vertical lines indicate the preferred-specification estimate for each scenario.

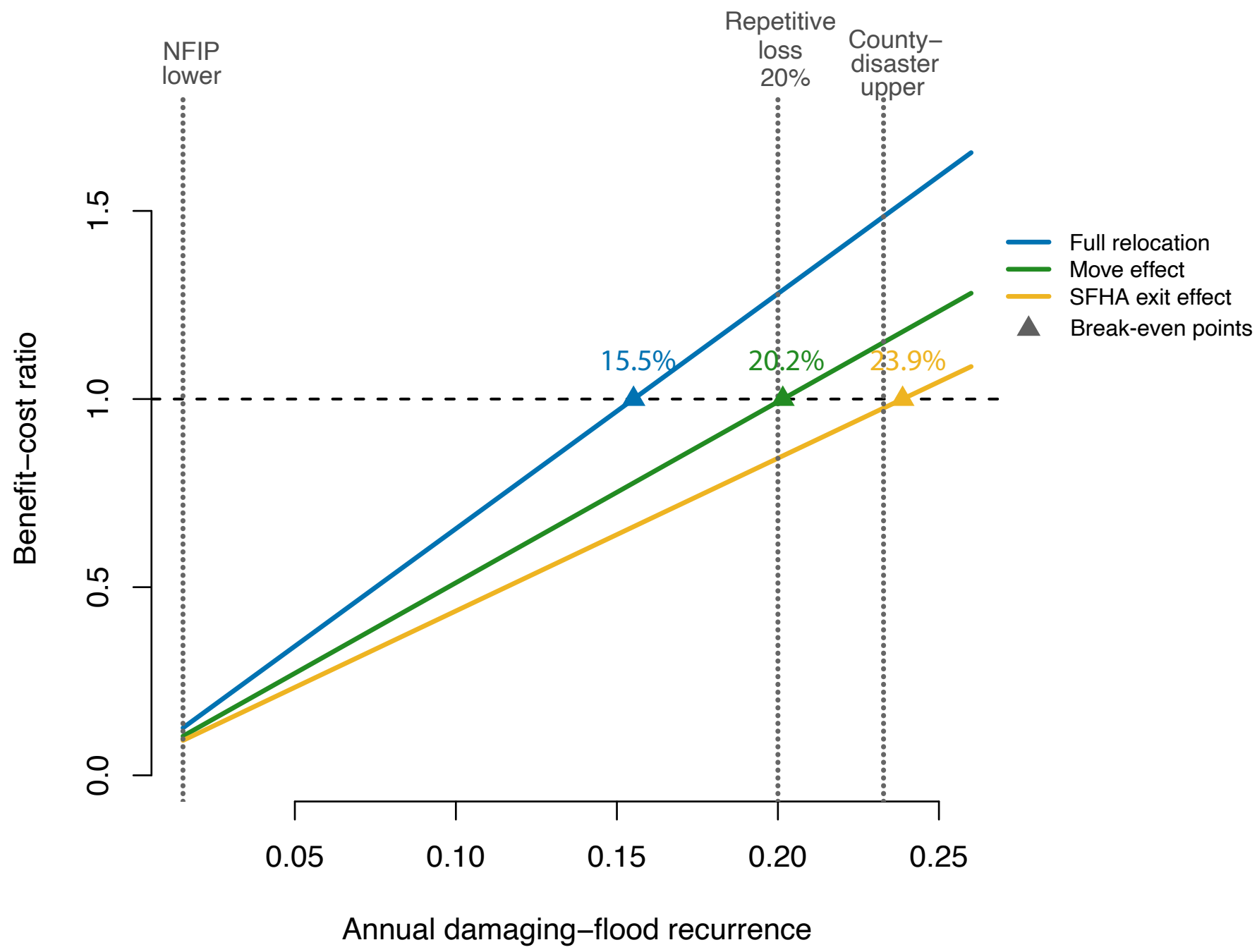

Figure S20: **Benefit–cost ratio as a function of assumed damaging-flood recurrence.** Deterministic benefit–cost ratios (preferred cost, disaster-assistance, and open-space amenity assumptions; 30-year horizon; 3% real discount rate) are plotted as a function of the assumed annual damaging-flood recurrence probability, shown separately for each of the three buyout scenarios (colored lines). Vertical dotted lines mark the three recurrence benchmarks used elsewhere in the analysis: the lower NFIP-based benchmark, the 20% repetitive-loss benchmark, and the upper county-level disaster-declaration benchmark. Triangles mark the recurrence probability at which each scenario's benefit–cost ratio equals one. The horizontal dashed line indicates a benefit–cost ratio of one.

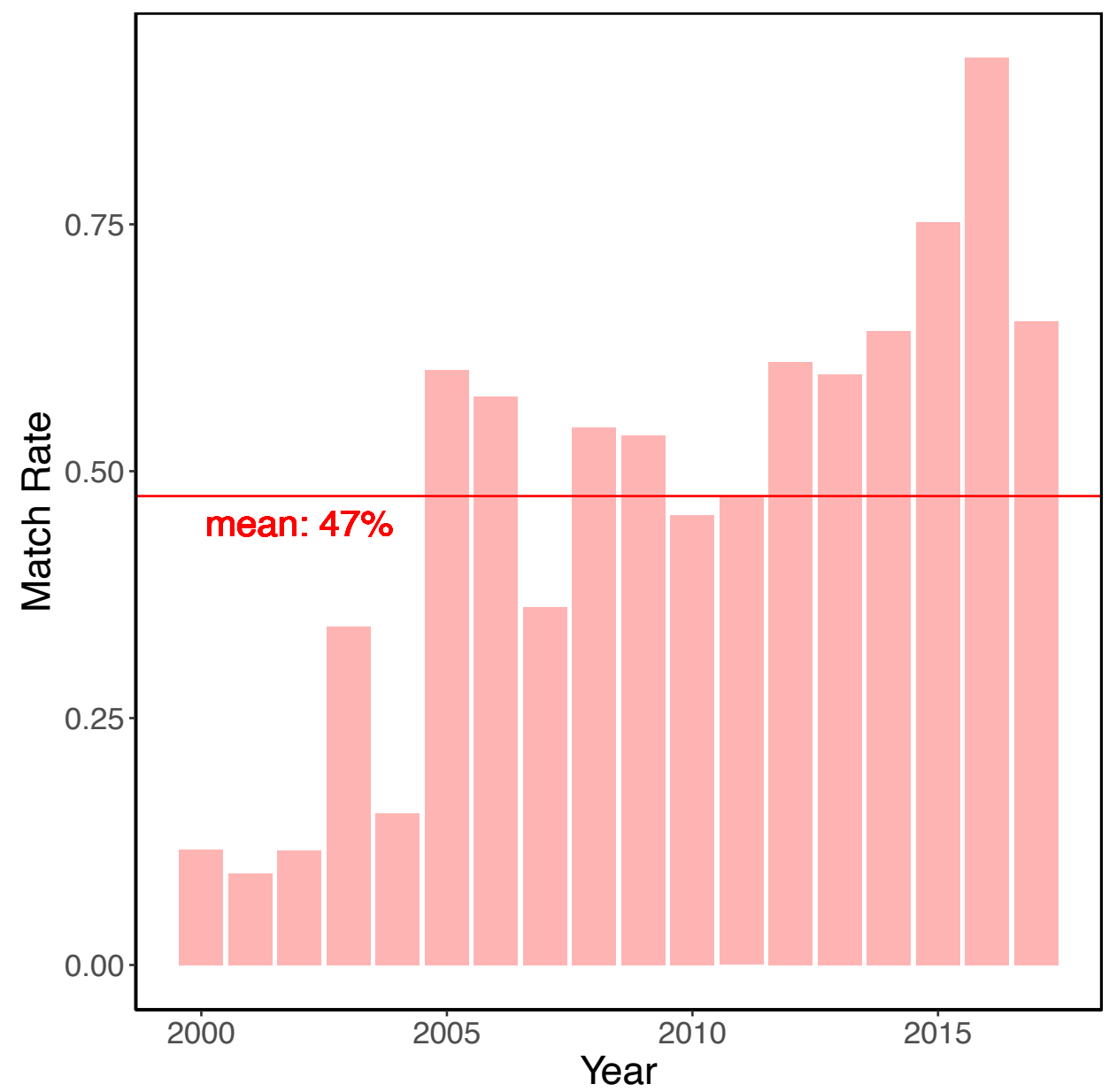

Figure S21: **Buyout (treated) address match rate over time**. Match rate between the NPR FEMA buyout data and the Infutor Address data, by year of the buyout, from 2000–2017 and the mean match rate across all years.

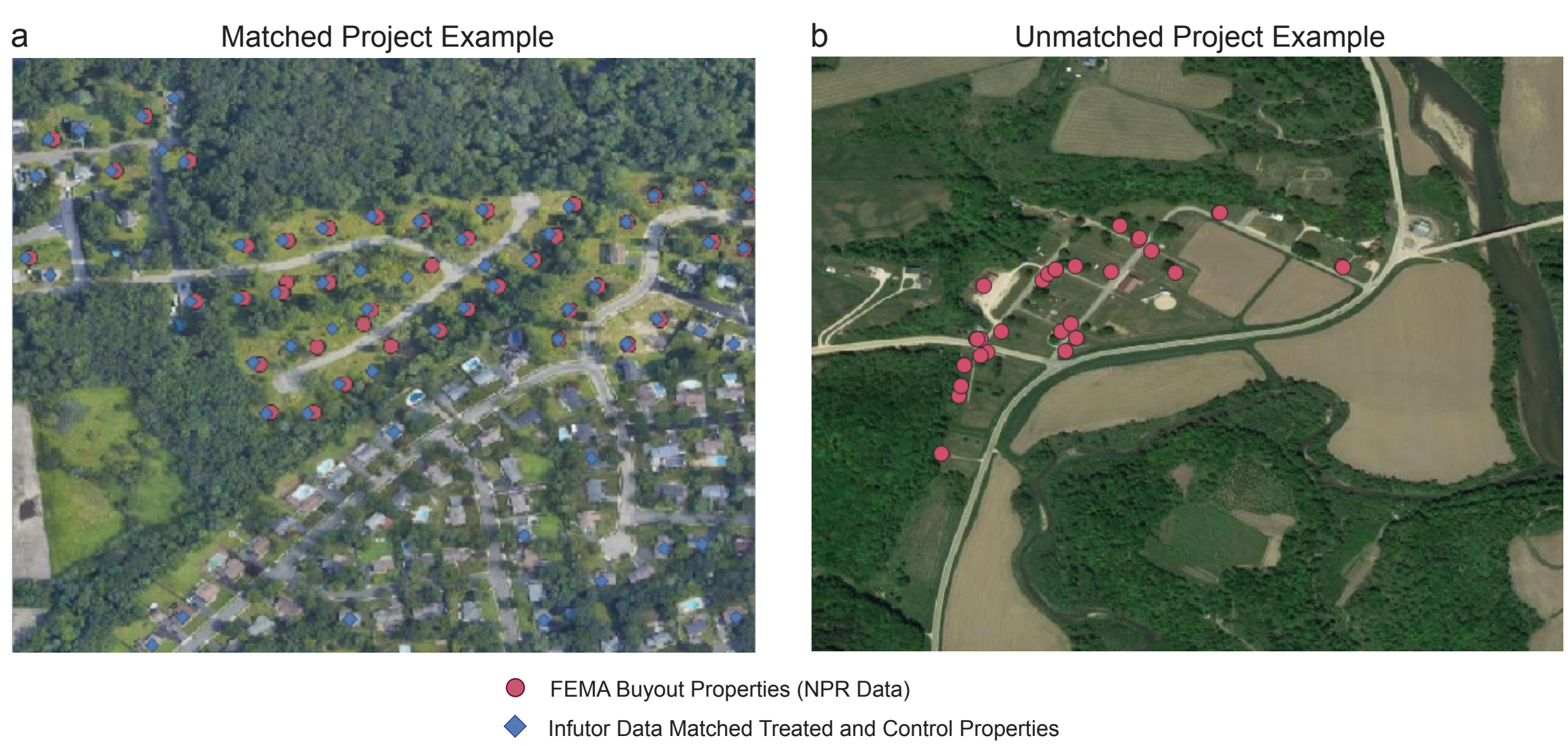

Figure S22: **Examples of matched and unmatched buyout projects.** (**a**) Example of a buyout project with successful matches between NPR FEMA addresses (red circles) and Infutor addresses (blue diamonds). (**b**) Example of a buyout project with no matched Infutor addresses. *Author-generated map created in ArcGIS using a Google Maps basemap. Basemap © Google.*

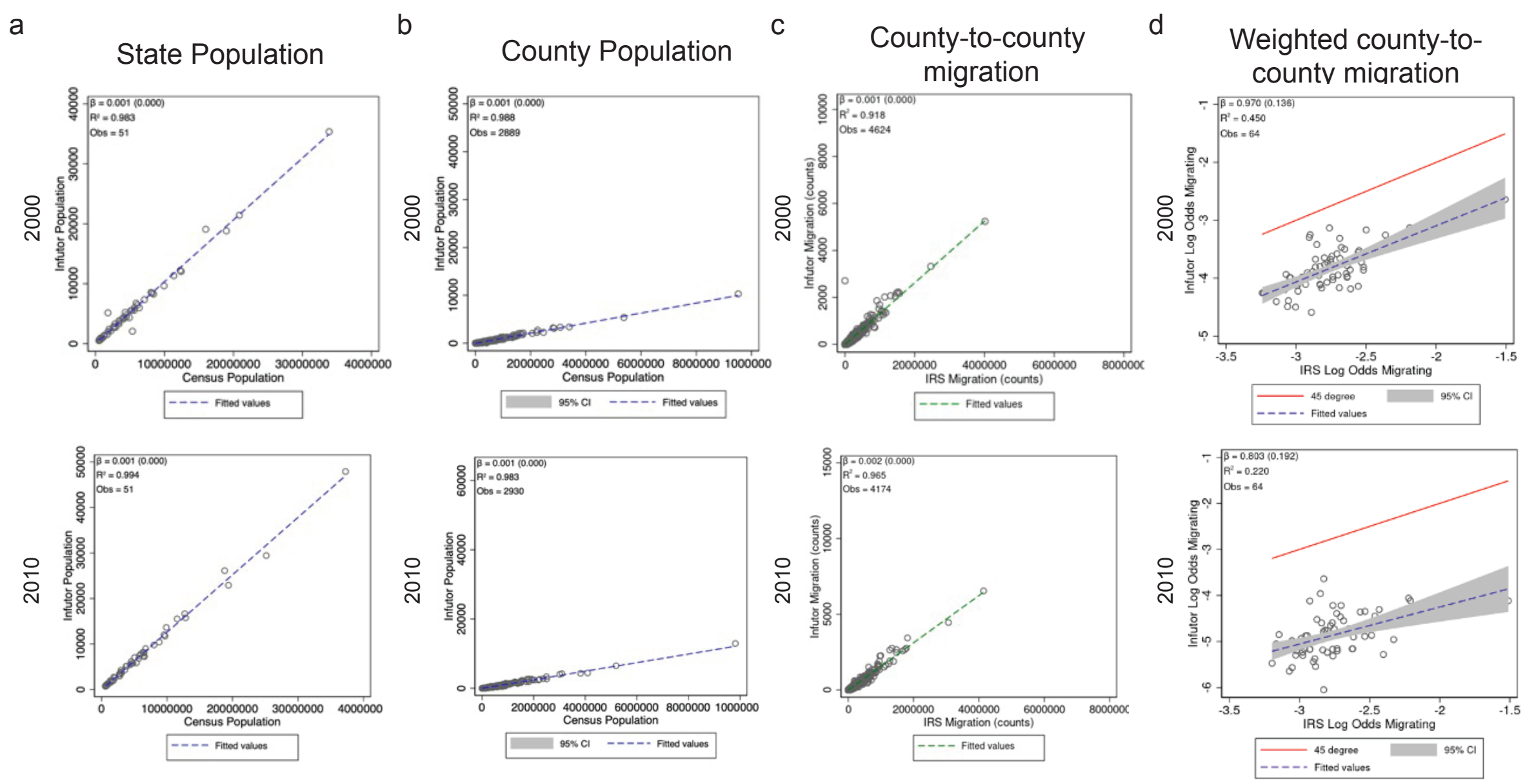

Figure S23: **Comparison of Infutor and public Census and IRS data**. (**a**) Comparison of Infutor state population (based on a 0.1% random sample) against Decennial Census population in 2000 and 2010 (N = 51, $R^2 > 0.98$, $\beta = 0.001$). (**b**) Comparison of Infutor county population counts (based on a 0.01% random sample) against Decennial Census population in 2000 and 2010 (N = 2930, $R^2 > 0.98$, $\beta = 0.001$). (**c**) Comparison of Infutor county-to-county out migration (based on a 0.01% random sample) against Internal Revenue Service's county-to-county migration counts in 2000 and 2010 (N = 4624, $R^2 > 0.91$, $\beta = 0.001$). (**d**) Population weighted log odds comparison of Infutor county-to-county out migration in Louisiana against Internal Revenue Service's county-to-county migration counts in 2000 (N = 54, $R^2 = 0.45$, $\beta = 0.970$) and 2010 (N = 64, $R^2 = 0.22$, $\beta = 0.803$) and the 45° line.

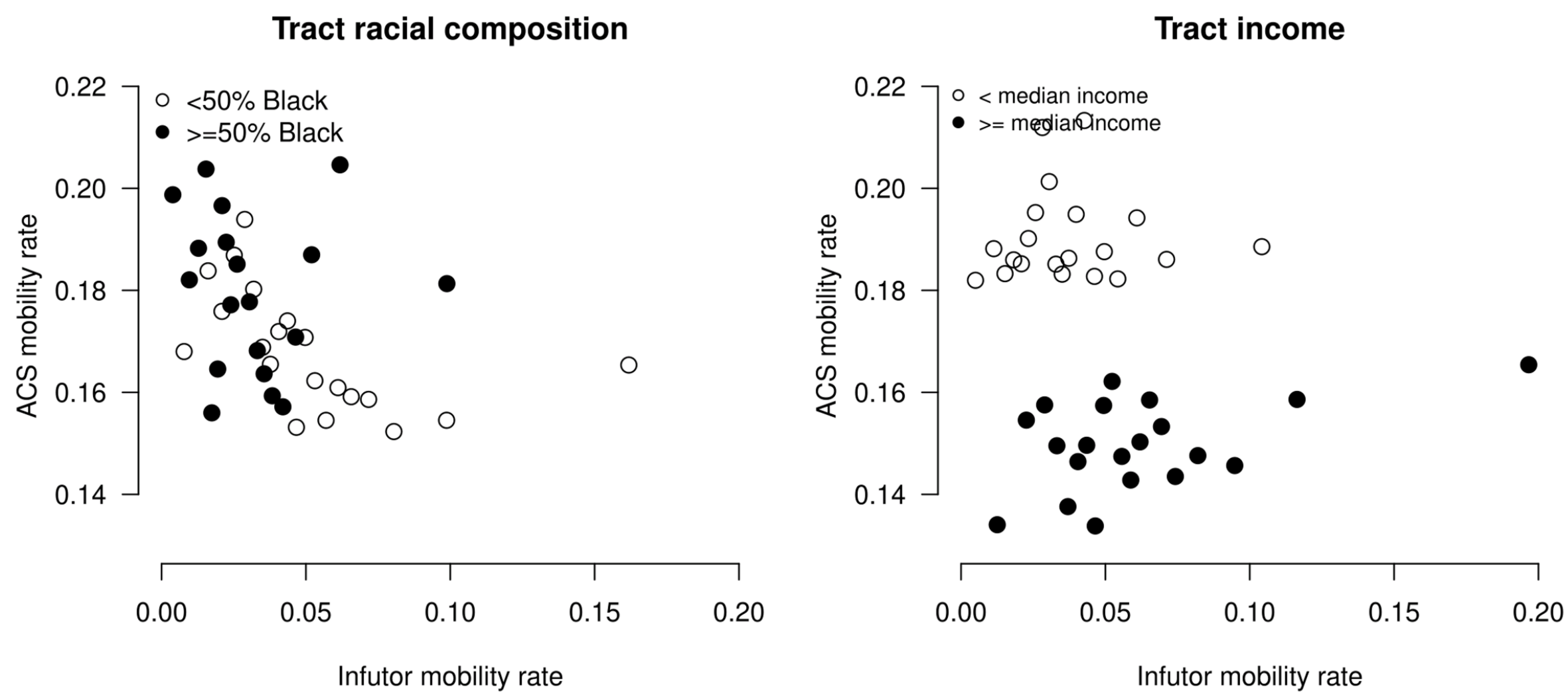

Figure S24: **Comparison of tract-level ACS and Infutor mobility rates across neighborhood demographic groups.** (a) Comparison of tract-level ACS and Infutor mobility rates among untreated individuals separately for census tracts with below- and above-50% Black population shares. (b) Comparison of tract-level ACS and Infutor mobility rates among untreated individuals separately for below- and above-median income census tracts. Points show binned averages of tract-level mobility rates. ACS mobility rates are measured using the share of residents reporting a different residence one year prior in the 2005–2009 American Community Survey. Infutor mobility rates are constructed from year-over-year address changes in the Infutor address panel.

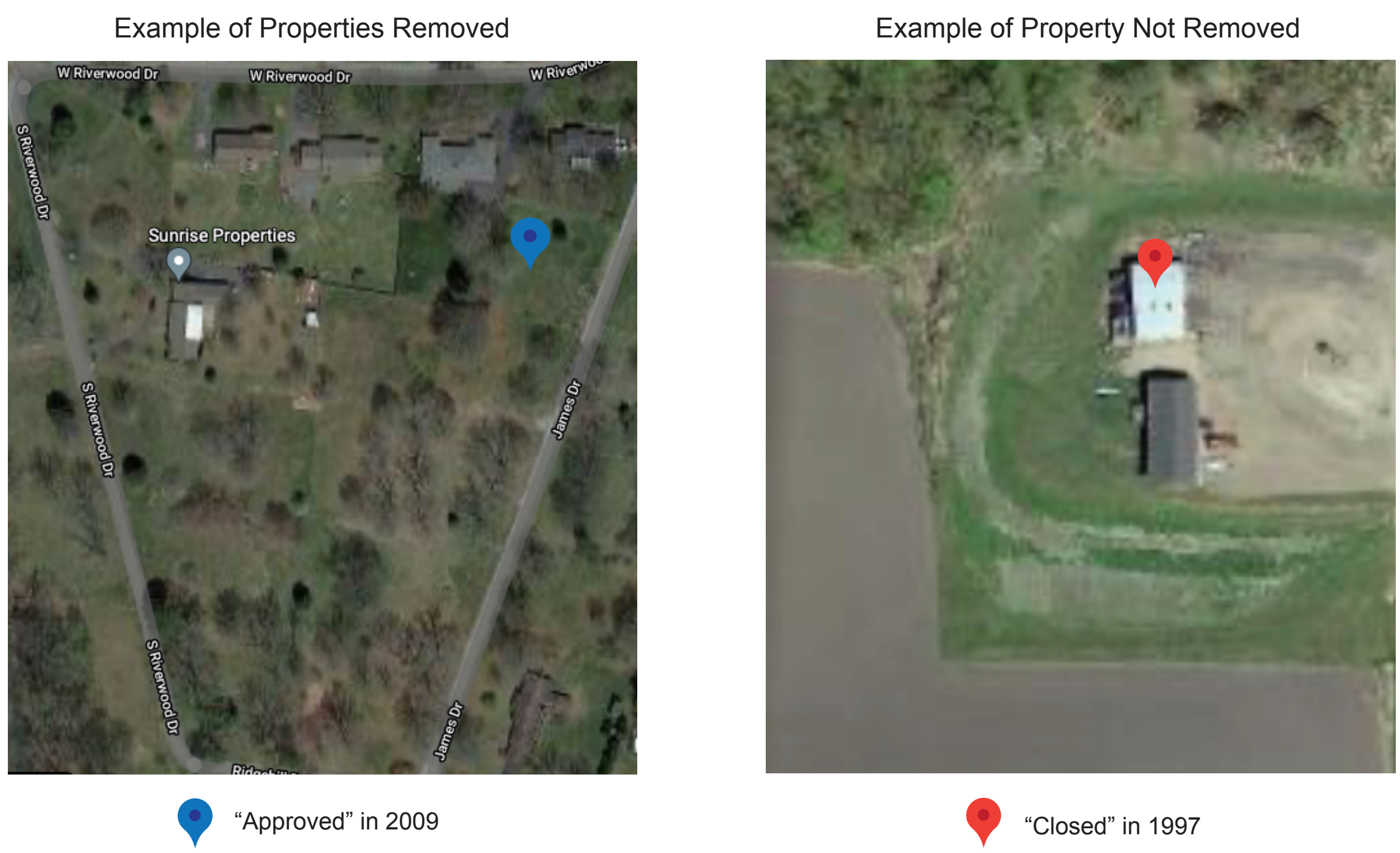

Figure S25: **Example satellite images of properties marked as buyouts.** (**a**) A property marked as a buyout with evidence of demolition. (**b**) A property marked as a buyout but with a structure still present decades later. *Image source: Google Maps. Map data © Google.*

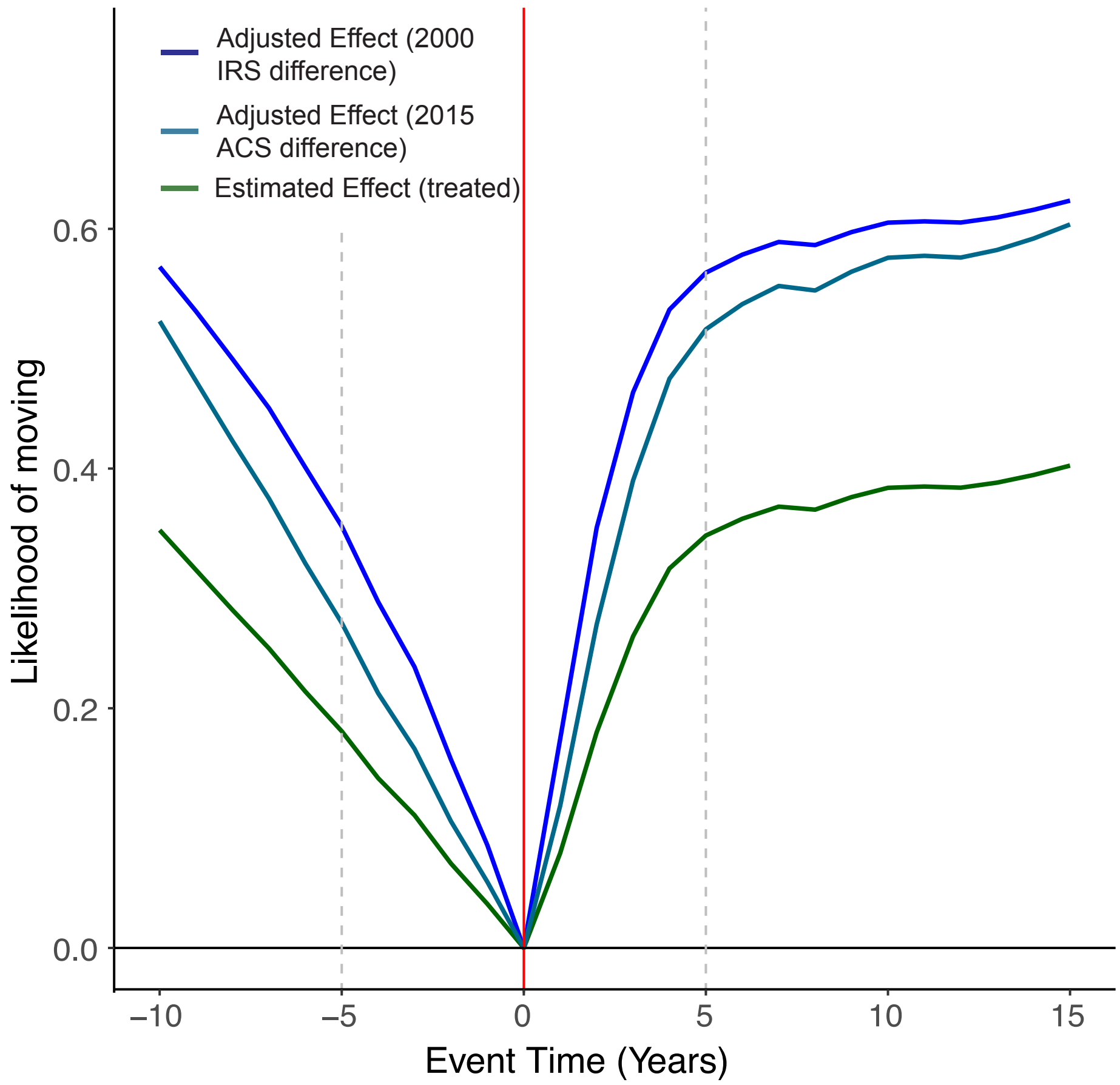

Figure S26: **Adjusted average likelihood of moving to a new address among buyout participants.** Green line shows the unadjusted likelihood of moving. Light blue line shows the adjustment based on the difference between Infutor move rates and the ACS 2015 "moved within 5 years" rate (Table S4). Dark blue line shows the adjustment based on the difference between Infutor move rates and the 2000 IRS county-to-county migration rate.

Program Name	Total Projects (Acquisition and Non-Acquisition)	No. Final Acquisition Properties	Federal Share Obligated	Avg. Cost Share
HMGP	22,732	43,619	$2.71B	74%
FMA	2,978	2,895	$505.86M	83%
PDM	4,534	2,213	$189.52M	75%
**Total**	**30,488**	**48,738**	**$3.42B**	**76%**

Table S1: **Total acquisitions and costs across major FEMA buyout programs.** Summary of the three largest FEMA programs that fund property acquisitions: (1) Hazard Mitigation Grant Program (HMGP); (2) Flood Mitigation Assistance (FMA); and (3) Pre-Disaster Mitigation (PDM). The table reports the total number of projects (including both acquisition and non-acquisition activities), the number of finalized property acquisitions ("No. Final Acquisition Properties"), the federal funds obligated ("Federal Share Obligated"), and the average federal cost share for each program.

	Buyout ZIP code				Buyout ZIP code and SFHA			
Variable	Control	Treated	p-value	Norm. Diff.	Control	Treated	p-value	Norm. Diff.
Census tract variables								
Property flood factor	2.04	2.73	0.00	-1.98	2.86	2.66	0.00	-0.60
Total population 2000	4,539	4,042	0.00	0.19	4,058	3,790	0.01	-0.15
Log total population 2000	8.33	8.32	0.00	0.16	8.24	8.20	0.12	0.11
Fraction Black	0.12	0.11	0.66	-0.02	0.22	0.21	0.36	-0.13
Fraction White	0.83	0.84	0.78	0.09	0.74	0.76	0.32	0.13
Fraction age 16+	0.49	0.49	0.76	-0.05	0.49	0.49	0.79	-0.18
Family income per capita ($)	21,757	21,233	0.26	0.03	21,261	21,390	0.74	-0.24
Fraction employed	0.48	0.43	0.09	0.03	0.47	0.48	0.43	-0.30
Fraction under 18	0.25	0.25	0.44	-0.09	0.27	0.28	0.07	0.00
Fraction over 65	0.14	0.15	0.45	0.02	0.13	0.13	0.96	0.00
Fraction college degree+	0.14	0.13	0.00	0.14	0.12	0.12	0.48	-0.17
Fraction HS / some college	0.47	0.48	0.00	-0.15	0.46	0.47	0.01	-0.05
Fraction less than HS	0.05	0.05	0.25	-0.08	0.06	0.05	0.00	0.27
Individual variables								
Pre-treatment move rate	0.07	0.06	0.00	-0.10	0.06	0.06	0.00	-0.05
Moved at least once (pre-treatment)	0.39	0.39	0.58	0.00	0.36	0.39	0.00	0.05
Unique pre-treatment addresses	1.65	1.64	0.13	-0.01	1.59	1.64	0.00	0.05
Observed years at same address	8.15	14.41	0.00	0.42	8.41	14.41	0.00	0.40
Years observed before treatment	10.85	17.49	0.00	0.45	10.87	17.49	0.00	0.44
VantageScore 4.0	671.49	669.16	0.75	-0.02	654.01	669.16	0.07	0.14
Total balance ($)	157,342	138,187	0.27	-0.06	135,842	138,187	0.90	-0.01
Bankcard balance ($)	36,197	33,235	0.30	-0.04	37,183	33,235	0.31	-0.05
Mortgage balance ($)	177,593	152,513	0.31	-0.10	168,614	152,513	0.55	-0.06
Baseline bankcard balance ($)	31,635	28,560	0.22	-0.05	31,297	28,560	0.42	-0.04
Credit card balance ($)	12,616	11,801	0.52	-0.03	13,628	11,801	0.35	-0.04
Credit available ($)	27,683	25,927	0.38	-0.04	33,391	36,565	0.46	-0.03
Has mortgage	0.33	0.32	0.64	-0.02	0.29	0.32	0.21	0.07

Table S2: **Baseline balance between buyout participants and comparison groups.** Means for treated and control groups and the p-values of their differences are shown for observable census-tract and individual-level credit characteristics measured prior to treatment. Two control groups are reported: (1) individuals living in the buyout ZIP code in the year of the buyout (“Buyout ZIP code”); and (2) individuals in the buyout ZIP code and in the Special Flood Hazard Area (“Buyout ZIP code and SFHA”). Normalized differences in means are calculated following the Imbens and Rubin (2016) normalization procedure. Smaller normalized differences indicate greater balance between treated and control groups.

**Individual-level financial variables**	**Buyout ZIP code**	**ZIP + SFHA**	**ZIP + SFHA + Flood Group (all)**	**ZIP + SFHA + Flood Group (excluding minimal)**
Credit card balance	-0.03	0.01	-0.012	0.01
Total balance	-0.06	0.06	-0.04	0.08
Bankcard balance	-0.04	-0.05	-0.06	0.04
Mortgage balance	-0.10	-0.06	-0.05	0.12
Credit available	-0.04	-0.03	0.02	0.07
Has mortgage	-0.02	0.07	0.11	0.24
VantageScore 4.0	-0.02	0.14	0.06	0.08
Mean absolute normalized difference	0.04	0.06	0.05	0.09

Table S3: **Normalized differences in means across alternative comparison groups for individual-level financial characteristics.** The table reports absolute normalized differences in means between treated households and alternative control groups for pre-treatment financial variables measured at event time $t = -1$. Smaller values indicate greater balance between treated and control households. Columns compare progressively more restrictive comparison groups: households within the same buyout ZIP code, households within the same ZIP code and Special Flood Hazard Area (SFHA), households within the same ZIP code, SFHA, and simplified flood-risk category, and the same specification excluding the "minimal" flood-risk group.

	Moved in within 5 years	Moved in within 10 years	Moved in within 15 years
ACS 2015	43%	-	71%
ACS 2010	45%	63%	-
Infutor (Treated)	18%	35%	50%
Infutor (SFHA Control)	18%	34%	50%
Percent Difference between Infutor and ACS	-149%	-79%	-43%
Adjusted Infutor (Treated) IRS 2010 error	62%	80%	88%
Adjusted Infutor (Treated) IRS 2000 error	35%	57%	71%

Table S4: **Comparing and adjusting estimated Infutor migration rates**. American Community Survey (ACS) 2015 and 2010 measures of move rates—moving to the current address within 5, 10, or 15 years—are compared to pre-buyout move rates derived from the matched Infutor address panel for buyout participants and the control group. Percent differences between ACS and Infutor migration rates are shown. Adjusted Infutor (treated) migration rates are computed using the gap in weighted log-odds of migrating between the Internal Revenue Service (IRS) county-to-county migration data (2010 and 2000) and Infutor migration rates (Fig. S23).

	m1	m2	m3	m4	m5	m6	m6 (SFHA)
treated	-0.016	-0.011	-0.007	-0.0011	0.034	-0.0044	-0.012
	(0.00)	(0.00)	(0.01)	(0.00)	(0.12)	(0.01)	(0.01)
5 years after buyout	0.116	0.114	0.112	0.111	0.11	0.094	0.09
	(0.00)	(0.00)	(0.00)	(0.00)	(0.00)	(0.00)	(0.00)
treated * 5 years after buyout	0.178	0.177	0.175	0.358	0.164	0.178	0.178
	(0.01)	(0.01)	(0.01)	(0.15)	(0.01)	(0.01)	(0.01)
cohort FE	no	yes	yes	yes	yes	yes	yes
state FE	no	yes	yes	yes	yes	yes	yes
state × cohort FE	no	yes	yes	yes	yes	yes	yes
county FE	no	no	yes	yes	yes	yes	yes
flood group FE	no	no	no	yes	yes	no	no
flood group interacted	no	no	no	no	yes	no	no
pre-move rate	no	no	no	no	no	yes	yes
N	1,382,226	1,356,244	1,356,244	1,356,244	1,356,244	1,356,244	120,904

Table S5: **Comparison of the main results across eight versions of the model.** Results from eight model specifications with different sets of fixed effects, all estimated using the control population living in the Special Flood Hazard Area (SFHA). Standard errors are shown in parentheses below point estimates. The table reports the key coefficients of interest: the effect of being treated ("Treated"), the effect five years after the buyout for the control group ("5 years after the buyout"), and the estimated main effect of the buyout program five years after the buyout relative to the control group ("Treated × 5 years after the buyout").

Parameter	Preferred	Low	High	Notes / Source
Property acquisition cost ($ per property)	87,160	7,776	325,583	Median positive acquisition expenditure from FEMA administrative buyout data; excludes zero-valued observations. (*40*)
Relocation assistance ($ per property)	0	0	5,000	Optional relocation assistance expenditure; sensitivity analysis only.
Demolition and site restoration cost ($ per property)	35,000	15,000	80,000	Includes demolition, environmental remediation, grading, and site restoration costs. (*18*)
Administrative and transaction cost share	0.15	0.10	0.25	Applied to the sum of acquisition, relocation, and demolition/site restoration costs; includes project administration, planning, environmental review, legal/title work, and project management. (*42*)
Deadweight cost of public funds	0.00	0.00	0.30	Marginal cost of public funds applied to total program cost; preferred value zero, varied in sensitivity analysis only.
Conditional building-and-contents loss ($ per damaging flood event)	45,917	4,911	55,629	Mean combined building-and-contents payment among paid NFIP claims (building or contents payment >0) for properties in FEMA A/V flood zones within buyout-sample ZIP codes. (*10*)
Damaging-flood recurrence probability (p_f)	0.20	0.0153	0.2328	Three fixed benchmarks evaluated in parallel, not a sampled distribution: lower bound from NFIP zone-weighted paid-claim incidence; 20% (repetitive-loss classification and preferred); upper bound from buyout-county flood disaster-declaration frequency. (*10, 41*)
Annual expected FEMA IHP disaster assistance ($ per property, conditional on a damaging flood event)	4,811	2,131	9,299	Mean/min/max of a 21-year per-property panel of Individuals and Households Program (IHP) payments. (*41*)
Open-space amenity co-benefit ($ per property-year)	250	0	2,000	Included in the preferred specification; accrues independent of whether a flood occurs. (*18*)
Real discount rate	3%	1%	7%	Preferred value follows U.S. Office of Management and Budget guidance. (*43*)
Benefit horizon (years)	30	10	30	Alternative assumptions regarding persistence of avoided future public expenditures.

Table S6: **Benefit–cost analysis assumptions and sensitivity ranges.** The table summarizes the preferred parameter values, sensitivity ranges, and data sources used in the benefit–cost analysis of FEMA-funded buyout programs. Preferred values are calibrated using FEMA administrative buyout expenditures, historical National Flood Insurance Program (NFIP) claims and FEMA Individual and Households Program (IHP) disaster assistance data, empirically estimated treatment effects on mobility and Special Flood Hazard Area (SFHA) exit, and assumptions drawn from the buyout literature. Low and high values are used in Monte Carlo simulations and one-at-a-time sensitivity analyses to characterize uncertainty regarding acquisition costs, implementation costs, disaster assistance, discounting, conditional building and content loss, and the persistence of avoided future public expenditures. All monetary values are expressed in real dollars on a per-property basis.

REFERENCES

1. K. J. Mach, A. R. Siders, Reframing strategic, managed retreat for transformative climate adaptation. *Science* **372**, 1294–1299 (2021).

2. H. Lauer, C. M. C. Chaves, E. Lorenzo, S. Islam, J. Birkmann, Risk reduction through managed retreat? Investigating enabling conditions and assessing resettlement effects on community resilience in Metro Manila. *Nat. Hazards Earth Syst. Sci.* **24**, 2243–2261 (2024).

3. A. Hsiao, Sea level rise and urban inequality. *AEA Pap. Proc.* **114**, 47–51 (2024).

4. H. T. N. Nguyen, *Vietnam - Emergency Flood Disaster Reconstruction Project: Resettlement Plan. Volume 2 of 7: Resettlement Action Plan – Binh Dinh Subproject* (tech. rep., World Bank, Vietnam, 2017), http://documents.worldbank.org/curated/en/766181498221627481.

5. J. R. Elliott, P. L. Brown, K. Loughran, Racial inequities in the federal buyout of flood-prone homes: A nationwide assessment of environmental adaptation. *Socius* **6**, 2378023120905439 (2020).

6. K. J. Mach, C. M. Kraan, M. Hino, A. R. Siders, E. M. Johnston, C. B. Field, Managed retreat through voluntary buyouts of flood-prone properties. *Sci. Adv.* **5**, eaax8995 (2019).

7. J. R. Elliott, Z. Wang, Managed retreat: A nationwide study of the local, racially segmented resettlement of homeowners from rising flood risks. *Environ. Res. Lett.* **18**, 064050 (2023).

8. Federal Emergency Management Agency, Hazard Mitigation Assistance Grants [accessed 1 March 2023]; www.fema.gov/grants/mitigation.

9. HUD Exchange, US Department of Housing and Urban Development, Community Development Block Grant Disaster Recovery (CDBG-DR) Disaster Recovery Buyout Program [accessed 1 March 2023]; www.hudexchange.info/programs/cdbg-dr/disaster-recovery-buyout-program/.

10. Federal Emergency Management Agency, OpenFEMA [accessed 22 June 2021]; www.fema.gov/about/reports-and-data/openfema.

11. J. Roback, Wages, rents, and the quality of life. *J. Polit. Econ.* **90**, 1257–1278 (1982).

12. J. R. Harris, M. P. Todaro, Migration, unemployment and development: A two-sector analysis. *Am. Econ. Rev.* **60**, 126–142 (1970).

13. J. E. Anderson, The gravity model. *Annu. Rev. Econ.* **3**, 133–160 (2011).

14. P. Kline, E. Moretti, People, places, and public policy: Some simple welfare economics of local economic development programs. *Annu. Rev. Econ.* **6**, 629–662 (2014).

15. US Department of Homeland Security Office of Inspector General, "FEMA needs to improve oversight and management of hazard mitigation grant program property acquisitions" (Report No. OIG-22-46, US Department of Homeland Security Office of Inspector General, 2022); www.oig.dhs.gov/sites/default/files/assets/2022-06/OIG-22-46-Jun22.pdf.

16. J. Gallagher, Learning about an infrequent event: Evidence from flood insurance take-up in the United States. *Am. Econ. J. Appl. Econ.* **6**, 206–233 (2014).

17. S. Miller, L. R. Wherry, D. G. Foster, The economic consequences of being denied an abortion. *Am. Econ. J. Econ. Policy* **15**, 394–437 (2023).

18. W. Curran-Groome, H. Haygood, M. Hino, T. K. BenDor, D. Salvesen, Assessing the full costs of floodplain buyouts. *Clim. Change* **168**, 3 (2021).

19. W. Curran-Groome, M. Hino, T. K. BenDor, D. Salvesen, Complexities and costs of floodplain buyout implementation. *Land Use Policy* **118**, 106128 (2022).

20. R. Jerch, M. E. Kahn, G. C. Lin, Local public finance dynamics and hurricane shocks. *J. Urban Econ.* **134**, 103516 (2023).

21. R. Arnott, Optimal city size in a spatial economy. *J. Urban Econ.* **6**, 65–89 (1979).

22. J. R. Bartle, K. A. Kriz, B. Morozov, Local government revenue structure: Trends and challenges. *J. Public Budg. Account. Financ. Manag.* **23**, 268–287 (2011).

23. T. Deryugina, D. Molitor, Does when you die depend on where you live? Evidence from Hurricane Katrina. *Am. Econ. Rev.* **110**, 3602–3633 (2020).

24. R. Chetty, N. Hendren, L. F. Katz, The effects of exposure to better neighborhoods on children: New evidence from the moving to opportunity experiment. *Am. Econ. Rev.* **106**, 855–902 (2016).

25. R. Chetty, N. Hendren, The impacts of neighborhoods on intergenerational mobility I: Childhood exposure effects. *Q. J. Econ.* **133**, 1107–1162 (2018).

26. J. Hinkel, D. Lincke, A. T. Vafeidis, M. Perrette, R. J. Nicholls, R. S. J. Tol, B. Marzeion, X. Fettweis, C. Ionescu, A. Levermann, Coastal flood damage and adaptation costs under 21st century sea-level rise. *Proc. Natl. Acad. Sci. U.S.A.* **111**, 3292–3297 (2014).

27. J. A. Church, N. J. White, Sea-level rise from the late 19th to the early 21st century. *Surv. Geophys.* **32**, 585–602 (2011).

28. Y. Hirabayashi, R. Mahendran, S. Koirala, L. Konoshima, D. Yamazaki, S. Watanabe, H. Kim, S. Kanae, Global flood risk under climate change. *Nat. Clim. Chang.* **3**, 816–821 (2013).

29. J. Howell, E. Korver-Glenn, Neighborhoods, race, and the twenty-first-century housing appraisal industry. *Sociol. Race Ethn.* **4**, 473–490 (2018).

30. A. M. Perry, J. Rothwell, D. Harshbarger, "The devaluation of assets in Black neighborhoods: The case of residential property," *The Brookings Institution*, 27 November 2018.

31. D. Aaronson, D. Hartley, B. Mazumder, The effects of the 1930s HOLC "redlining" maps. *Am. Econ. J. Econ. Policy* **13**, 355–392 (2021).

32. R. Young, S. Hsiang, Mortality caused by tropical cyclones in the United States. *Nature* **635**, 121–128 (2024).

33. C. Fu, J. Gregory, Estimation of an equilibrium model with externalities: Post-disaster neighborhood rebuilding. *Econometrica* **87**, 387–421 (2019).

34. C. A. Taylor, H. Druckenmiller, Wetlands, flooding, and the Clean Water Act. *Am. Econ. Rev.* **112**, 1334–1363 (2022).

35. R. T. Woodward, Y.-S. Wui, The economic value of wetland services: A meta-analysis. *Ecol. Econ.* **37**, 257–270 (2001).

36. US Census Bureau, Decennial Census Official Publications: 2000 [accessed 24 June 2024]; www.census.gov/programs-surveys/decennial-census/decade/decennial-publications.2000.html.

37. US Census Bureau, Decennial Census Official Publications: 2010 [accessed 24 June 2024]; www.census.gov/programs-surveys/decennial-census/decade/decennial-publications.2010.html.

38. US Census Bureau, Decennial Census Official Publications: 2020 [accessed 24 June 2024]; www.census.gov/programs-surveys/decennial-census/decade/decennial-publications.2020.html.

39. G. W. Imbens, D. B. Rubin, *Causal Inference for Statistics, Social, and Biomedical Sciences: An Introduction* (Cambridge University Press, 2015). 10.1017/CBO9781139025751.

40. R. Benincasa, "Search the thousands of disaster buyouts FEMA didn't want you to see," *National Public Radio (NPR)* (2019); www.npr.org/2019/03/05/696995788/search-the-thousands-of-disaster-buyouts-fema-didnt-want-you-to-see.

41. Federal Emergency Management Agency, OpenFEMA Data Sets: Hazard Mitigation (Federal Emergency Management Agency, 2026); www.fema.gov/about/openfema/data-sets.

42. Office of Management and Budget, Discount Rates for Cost-Effectiveness Analysis of Federal Programs Federal Register 87, no. 11085 (24 May 2022): 31585.

43. US Census Bureau, TIGER/Line Shapefiles [accessed June 2026] (2020); www.census.gov/geographies/mapping-files/time-series/geo/tiger-line-file.html.

44. R. Young, Replication data for Does Government-Assisted Relocation Help Households, Zenodo (2026); 10.5281/zenodo.22048109.

45. R. Young, Replication package for "Does Government-Assisted Relocation Help Households," version accompanying the *Science Advances* submission, GitHub [accessed June 2026] (2026); https://github.com/rachelmyoung/buyout.

46. R. Diamond, T. McQuade, F. Qian, The effects of rent control expansion on tenants, landlords, and inequality: Evidence from San Francisco. *Am. Econ. Rev.* **109**, 3365–3394 (2019).

47. B. J. Asquith, E. Mast, D. Reed, Local effects of large new apartment buildings in low-income areas. *Rev. Econ. Stat.* **105**, 359–375 (2023).

48. F. Qian, R. Tan, "The effects of high-skilled firm entry on incumbent residents" (working paper no. 21-042, Stanford Institute for Economic Policy Research, 2021). http://dx.doi.org/10.2139/ssrn.3982760.

49. J. B. Mathew Hauer, IRS county-to-county migration data, 1990–2010. *Demogr. Res.* **40**, 1153–1166 (2019).

50. Internal Revenue Service, SOI Tax Stats—Migration Data [accessed 24 June 2021] (2020); www.irs.gov/statistics/soi-tax-stats-migration-data.

51. US Census Bureau, American Community Survey [accessed 24 October 2021] (2021); www.census.gov/programs-surveys/acs/.
